# WHO-led consensus statement on vaccine delivery costing: process, methods, and findings

**DOI:** 10.1186/s12916-022-02278-4

**Published:** 2022-03-08

**Authors:** Ann Levin, Laura Boonstoppel, Logan Brenzel, Ulla Griffiths, Raymond Hutubessy, Mark Jit, Vittal Mogasale, Sarah Pallas, Stephen Resch, Christian Suharlim, Karene Hoi Ting Yeung

**Affiliations:** 1 Levin & Morgan LLC, Bethesda, USA; 2 ThinkWell, Geneva, Switzerland; 3 Bill & Melinda Gates Foundation, Seattle, USA; 4 UNICEF, New York, USA; 5 Department of Immunization, Vaccines and Biologicals, World Health Organization, Geneva, Switzerland; 6 London School of Hygiene & Tropical Medicine, London, UK; 7 International Vaccine Institute, Seoul, South Korea; 8 Centers for Disease Control and Prevention, Atlanta, USA; 9 Harvard T.H. Chan School of Public Health, Boston, USA

**Keywords:** Delivery cost, Vaccine, Immunization, Consensus statement, Costing, Guideline

## Abstract

**Background:**

Differences in definitions and methodological approaches have hindered comparison and synthesis of economic evaluation results across multiple health domains, including immunization. At the request of the World Health Organization’s (WHO) Immunization and Vaccines-related Implementation Research Advisory Committee (IVIR-AC), WHO convened an ad hoc Vaccine Delivery Costing Working Group, comprising experts from eight organizations working in immunization costing, to address a lack of standardization and gaps in definitions and methodological guidance. The aim of the Working Group was to develop a consensus statement harmonizing terminology and principles and to formulate recommendations for vaccine delivery costing for decision making. This paper discusses the process, findings of the review, and recommendations in the Consensus Statement.

**Methods:**

The Working Group conducted several interviews, teleconferences, and one in-person meeting to identify groups working in vaccine delivery costing as well as existing guidance documents and costing tools, focusing on those for low- and middle-income country settings. They then reviewed the costing aims, perspectives, terms, methods, and principles in these documents. Consensus statement principles were drafted to align with the Global Health Cost Consortium costing guide as an agreed normative reference, and consensus definitions were drafted to reflect the predominant view across the documents reviewed.

**Results:**

The Working Group identified four major workstreams on vaccine delivery costing as well as nine guidance documents and eleven costing tools for immunization costing. They found that some terms and principles were commonly defined while others were specific to individual workstreams. Based on these findings and extensive consultation, recommendations to harmonize differences in terminology and principles were made.

**Conclusions:**

Use of standardized principles and definitions outlined in the Consensus Statement within the immunization delivery costing community of practice can facilitate interpretation of economic evidence by global, regional, and national decision makers. Improving methodological alignment and clarity in program costing of health services such as immunization is important to support evidence-based policies and optimal resource allocation. On the other hand, this review and Consensus Statement development process revealed the limitations of our ability to harmonize given that study designs will vary depending upon the policy question that is being addressed and the country context.

**Supplementary Information:**

The online version contains supplementary material available at 10.1186/s12916-022-02278-4.

## Background

Immunization has been shown to provide a high return on investment across low- and middle-income countries [1]. Nevertheless, disparities in immunization access persist within and between countries. With the launch of the new Immunization Agenda 2030 [2], many low- and middle-income countries (LICs and MICs) are considering introducing new vaccines or vaccine-related technologies, life-course immunization programs, and improving the effectiveness and efficiency of their immunization programs. To determine the feasibility of doing so, estimation of vaccine procurement and delivery costs is of considerable interest to policymakers, program managers, researchers, and other stakeholders concerned with improving immunization programs. In particular, results from delivery cost studies can help countries in decision-making and planning on introducing new infant and life-course vaccines and technologies, preparation of budgets and financing for rollout of vaccines, and evaluation of alternative service delivery approaches.

Recent reviews of immunization delivery cost literature identified a lack of standardization in methods and reporting, making cross-study comparison and synthesis difficult [3]. These discrepancies limit the interpretability and utility of immunization cost study evidence for immunization program decision-making. In light of these challenges, the World Health Organization (WHO) Immunization and Vaccines-related Implementation Research Advisory Committee (IVIR-AC) recommended at their March 2018 meeting that the WHO Guidance on Vaccine Delivery Costing be updated [4]. An ad hoc Working Group comprising vaccine delivery costing experts^1^ was therefore convened by the WHO secretariat to review guidance documents and tools on vaccine delivery costing, focused on low- and middle-income country settings. This initial review found that several groups were already developing methodological guidance to address the disparate definitions and approaches in the field, which partly address the original IVIR-AC’s request. In March 2019, IVIR-AC modified its request to instead review guidance documents and costing tools, assess their similarities and differences, and identify gaps in guidance [5]. In addition, the Working Group recommended that a Consensus Statement be developed to harmonize the differences in costing terminology and principles for groups working in vaccine delivery costing.

For the purpose of this paper, vaccine delivery costing is defined as “costs associated with delivering immunizations to target populations, exclusive of vaccine procurement costs” [6].

This paper describes the history and process involved to develop the Consensus Statement on Vaccine Delivery Costing, the methods used and the findings of the review of guidance documents and costing tools, and terms and principles as well as recommendations agreed upon by the Working Group.

## Methods

### Process of developing the consensus statement

**Fig. 1:**
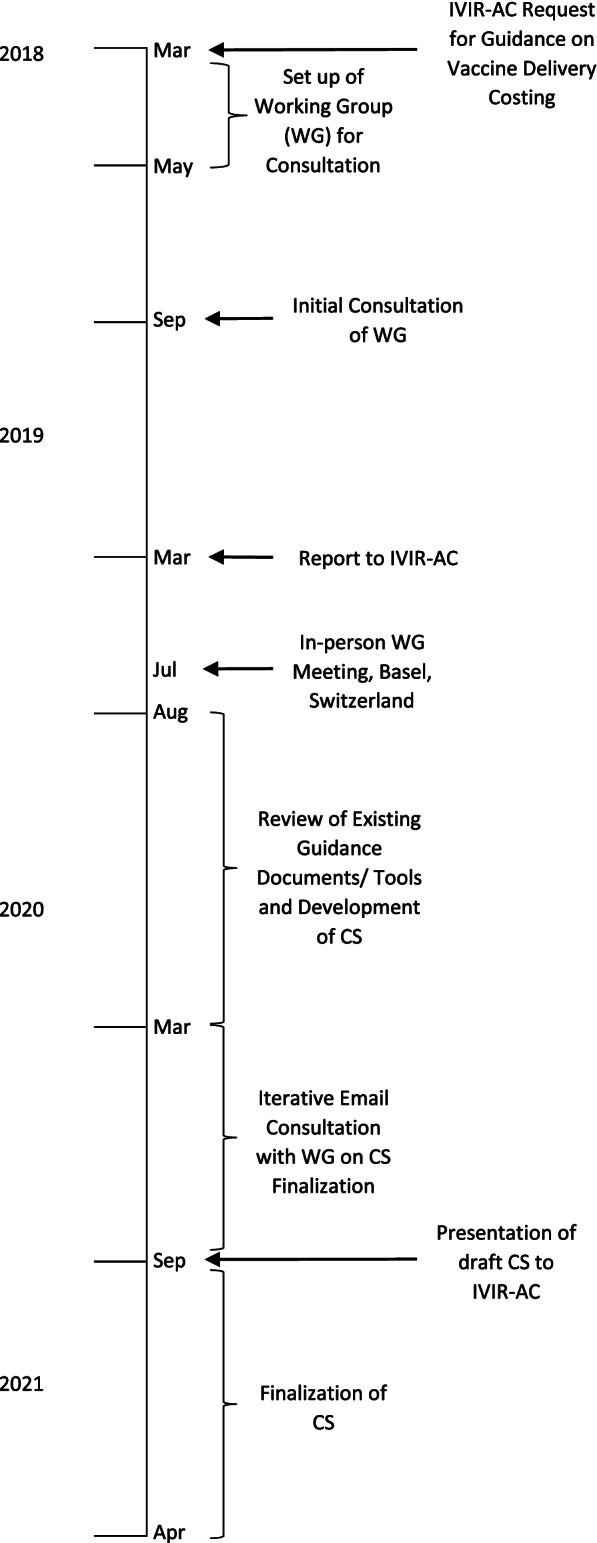
Timeline of the consultation process to develop a Consensus Statement (CS) on vaccine delivery costs

The consultation process of coming to agreement on a Consensus Statement included setting up a time-limited Working Group of staff of organizations working in vaccine delivery costing (who are also the authors of this paper), conducting a review of guidance documents, costing tools, and other documents and the costing terms, methods, and principles used in these, agreeing upon the costing terminologies and principles, making recommendations to harmonize their differences, writing the text of the Consensus Statement and Annexes (Additional file 1). Figure 1 shows a timeline of the meetings and activities that led to the development of the Consensus Statement.

In March 2018, the IVIR-AC initiated the process and requested that WHO update its guidance for conducting vaccine delivery costing in LICs and MICs so that methods used in costing tools and guidance documents could be standardized among WHO and other organizations.

As a follow-up, the WHO secretariat set up a Working Group of Experts comprising staff of organizations conducting research and policy advice on vaccine delivery costing in LICs and MICs to ensure that no parallel efforts were taking place. The initial Working Group comprised of technical experts from the Bill & Melinda Gates Foundation (BMGF), International Vaccine Institute (IVI), Levin & Morgan LLC, UNICEF, and WHO, and two members of IVIR-AC. The group noted that there are several ongoing workstreams conducting cost studies and developing guidance documents and/or costing tools, with different purposes and approaches to costing. In addition, some of these workstreams had developed guidance documents specific to their approach, which were already in the public domain. Thus, a review of these would be required to determine if an additional vaccine delivery costing guidance would be necessary. The Working Group also suggested that a presentation be made at the IVIR-AC meeting in March 2019 to present the findings on the different workstreams to determine the next steps.

In March 2019, the WHO team and the BMGF-funded ThinkWell project (Immunization Costing Action Network [ICAN]) presented to IVIR-AC on findings from the discussion with the Working Group [2].^2^ IVIR-AC recommended that an in-person workshop meeting be held with other groups working on vaccine delivery costing so that a consensus could be reached on the best way to standardize costing terms and principles.

In July 2019, WHO and the BMGF convened a meeting with eleven experts from different organizations and institutions in immunization economics during an International Health Economics Association (iHEA) meeting in Basel, Switzerland. The Working Group was expanded to include technical experts from other organizations involved in vaccine delivery costing such as Harvard (Expanded Programme on Immunization Costing [EPIC] studies) and the United States Centers for Disease Control and Prevention (CDC). Based on the subject matter knowledge and professional experience of the Working Group members, the different purposes of the workstreams were discussed and a matrix of costing tools listing out the characteristics of each was created. The Working Group agreed that it would be useful to develop a Consensus Statement that presents the different purposes of each workstream, a review of existing vaccine delivery costing guidance documents and tools, and agreed-upon costing terms and principles.

As a follow-up from the meeting in Basel, from August 2019 to March 2020, an analysis of guidance documents and tools was conducted for each of the four workstreams identified by the Working Group. The Group identified similarities and differences in costing methods, terms and principles among the approaches and in guidance documents, and gaps where further guidance was needed.

Based on these findings, the WHO team developed a proposed draft Consensus Statement report with recommendations for costing terms and principles that could be adhered to for future vaccine delivery costing work and accompanying annexes that summarized the findings from the review on costing terms, costing principles, and methods for vaccine delivery costing. After extensive consultation within the Working Group and several rounds of written revisions to reach consensus on the statement, the findings and recommendations were presented to IVIR-AC in September 2020. The IVIR-AC commended the process to create the Consensus Statement (Additional file 1) [7].

### Review of vaccine delivery costing guidance documents and tools

The first step was to conduct a landscape analysis of the organizations involved in vaccine delivery costing and their workstreams, and the available guidance documents and tools on vaccine delivery costs. This landscape analysis was conducted through discussions between the Working Group members during teleconferences and an in-person meeting as well as internet searches of websites of organizations working in the field (e.g., ICAN, Immunization Economics) between August 2019 and March 2020. It was not a systematic literature review and did not aim to include general health service costing tools and guidance documents beyond those with known use for costing immunization in LICs and MICs. However, the analysis built on the recent systematic review and reporting guidance for immunization costing studies conducted by some working group member organizations [3].

The second step was to compare the characteristics of the guidance documents and tools for immunization costing identified in terms of (1) how costing terms were defined in the guidance documents and costing tools; (2) whether data collection, sampling, and analysis were described in the guidance documents; and (3) whether costing principles were specified in guidance documents.

To review the costing terms in the guidance documents, the definitions were extracted from the source documents and entered into a table so that similarities and differences could be compared qualitatively and recommendations could be made for harmonized definitions for key terms. The costing principles and the guidance text, including on data collection, sampling, and analysis, were also compared and entered into a table by workstreams to assess the similarities, differences, and gaps. To do so, the costing principles in the guidance documents were compared to the ones in the checklist in the Global Health Cost Consortium (GHCC) [8] that has become a normative reference standard for global health costing work. These principles are similar to those found in the CHEERS checklist [9]. Recommendations were then made for harmonized principles in the Consensus Statement.

## Results

### Existing immunization delivery costing workstreams

**Fig. 2:**
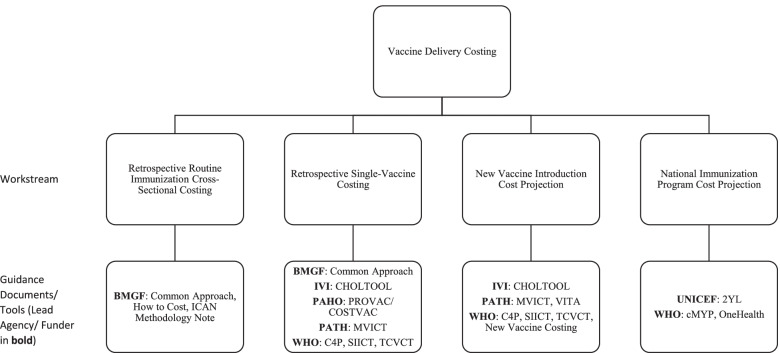
Major current workstreams in vaccine delivery costing identified by the Working Group. Note: 2YL, 2nd Year of Life; BMGF, Bill & Melinda Gates Foundation; C4P, Cervical Cancer Prevention and Control Costing; CDC, United States Centers for Disease Control and Prevention; CHOLTOOL, Oral Cholera Vaccine Costing Tool; cMYP, comprehensive multi-year plan; EPIC, Expanded Programme on Immunization Costing; ICAN, Immunization Costing Action Network; IVI, International Vaccine Institute; MVICT, Malaria Vaccine Immunization Costing Tool; SIICT, Seasonal Influenza Immunization Costing Tool; TCVCT, Typhoid Conjugate Vaccine Costing Tool; VTIA, Vaccine Technology Costs and Health Impact Assessment Tool; WHO, World Health Organization

The Working Group identified four major current workstreams on vaccine delivery costing in LICs and MICs. These include the following: (1) retrospective routine immunization (multiple vaccines) cross-sectional costing, (2) retrospective single-vaccine costing, (3) new vaccine introduction cost projection, and (4) national immunization program cost projection (Fig. 2). Although the workstreams had involvement from particular organizations at the time of the review, they are defined by their different objectives and corresponding methodologies and constitute a typology of immunization delivery costing work to which other organizations and practitioners beyond those listed contribute.

The first workstream is focused on estimating retrospective (i.e., already incurred) routine immunization cross-sectional costs of service delivery units at a single point in time for multiple vaccines delivered through the routine immunization program. These analyses focus on estimating routine immunization costs incurred at the facility, district, and higher administrative levels in the health system. Such analyses typically estimate unit costs (cost per dose, cost per person, or cost per fully immunized person [FIP]). Some examples of this work include the EPIC studies [10] and other work by institutes, such as the Harvard T.H. Chan School of Public Health, Wits University, Curatio Foundation, PAHO, ThinkWell, UNICEF, Johns Hopkins University, and PATH. The objectives of research within this workstream are to develop benchmarks for costs to be used in future studies, to analyze variation in unit costs, and to compare the findings with data from other costing studies [4].

The second workstream is to estimate retrospective costs for a specific vaccine or campaign, typically using incremental costing. That is, it usually aims to measure the value of additional resources employed to introduce a new vaccine or conduct a vaccination campaign. This is often done through data collection at a single point in time (post-campaign or post-introduction) with reference to documents and recall by key informants to estimate which resource use was specifically incremental. Examples of such studies include those conducted by groups such as EPIC, ThinkWell, CDC, and IVI. This workstream includes retrospective cost studies of vaccine implementation using vaccine-specific costing tools (e.g., Cervical Cancer Prevention and Control Costing [C4P], Oral Cholera Vaccine Costing Tool [CHOLTOOL], Malaria Vaccine Immunization Costing Tool [MVICT], Seasonal Influenza Immunization Costing Tool [SIICT], and Typhoid Conjugate Vaccine Costing Tool [TCVCT]). These studies yield results that will assist countries with comparing budgeted amounts to actual implementation resource use, budgeting for future immunization activities, and conducting cost-effectiveness analyses that compare the incremental resource use for a specific vaccine introduction or campaign with its incremental health impacts.

The third workstream is focused on estimating new vaccine introduction costs through projection of the value of resources or ingredients (e.g., time, equipment, training, and vaccines) needed for vaccine introduction, typically using incremental costing for a specific period. Data for these analyses are obtained through interviews with program managers and facility visits to obtain current information on personnel time, supplies, equipment, and other resources as well as retrospective cost data from other vaccine introduction. These analyses are often conducted using costing tools, including some of the same tools used for retrospective single-vaccine costing (e.g., C4P, CHOLTOOL, MVICT, SIICT, and TCVCT). These studies produce cost estimates that will assist countries with planning and decision-making on new vaccines during the introduction period.

The fourth workstream is projection of immunization program costs. Some costing tools used to produce these estimates include the comprehensive multi-year plan (cMYP), 2nd Year of Life (2YL), and OneHealth tool where the activities of a national program and related cost is entered for a baseline year and then the future years are projected. These analyses are an integral part of strategic planning for budgeting and resource mobilization over a specific period of time such as 5 years. Whereas work under the first three workstreams may produce estimates of financial, economic, or undepreciated financial costs, projections under the fourth workstream are intended to estimate undepreciated financial costs (i.e., undiscounted monetary outlays).

In practice, projects may combine elements of multiple workstreams (e.g., retrospective single vaccine costing in one country may be used to help inform estimates of new vaccine introduction costs for a different vaccine or delivery strategy).

### Existing guidance documents on vaccine delivery costing

Table 1 shows the nine guidance documents on vaccine delivery costing identified by the Working Group. Some of these provide guidance for more than one type of costing. Three are for estimation of retrospective routine immunization cross-sectional costs, five are for estimation of retrospective single-vaccine costs, five are for projection of new vaccine introduction costs, and one for projection of immunization program costs. The list of costing tools for vaccine delivery identified by the Working Group is shown in Additional file 1: Table A2b.

**Table 1:** List of guidance documents on vaccine delivery costing identified and reviewed by Working Group

Developer	Guidelines	Publication year	Target Interventions	Purposes	Workstream(s)^a^
EPIC	Common approach for the costing and financing analyses of routine immunization and new vaccine introduction costs [11]	2013	Existing and new vaccine programs	Methods for data collection for routine immunization programs and new vaccine introduction (including delivery costs) and financial flows	(1) (2)
EPIC	How to cost immunization programs - a practical guide on primary data collection and analysis [12]	2020	Existing and new vaccine programs	Practical guidance on how to conduct a facility-based exercise on immunization program costs, including sampling and analytical techniques	(1)
Global Health Cost Consortium (GHCC)	GHCC Reference Case [8]	2017	Health interventions in general	Improve quality of cost estimates	Not specific to a workstream
ICAN	Methodology note for systematic review, cost catalog, and analytics [6]	2019	Immunization delivery costs	Designed for users of data, including national and sub-national planners and policymakers, researchers, and international partners supporting country immunization and health system policy, planning, and financing	(1) (2)
IVI/WHO	CHOLTOOL User Guide [13]	2015	Cholera-specific vaccination programs, including campaigns	Instructions for users of costing tools	(2) (3)
WHO	Guidelines for estimating costs of introducing new vaccines into the national immunization system [14]	2002	New vaccine programs	Assist countries in planning for introduction of new vaccines	(3)
WHO	C4P tool: HPV Vaccination Module User Guide [15]	2012-2019	HPV vaccination programs	Instructions for users of costing tool	(2) (3)
WHO	cMYP Costing and Financing Tool User Guide [16]	2014	Immunization Program Costs	Instructions for users of costing tool	(3) (4)
WHO	Flutool plus (SIICT): introduction planning and costing [17]	2017	Seasonal influenza vaccination, including campaigns	Instructions for users of costing tool	(2) (3)

^a^(1) = retrospective routine immunization cross-sectional costing; (2) = retrospective single-vaccine costing; (3) = new vaccine introduction cost projection; (4) = national immunization program cost projection. Note: This table leaves out a few guidance documents (WHO 1994 and WHO 2019 listed in the Consensus Statement Annexes (Additional file 1: Table A2a)) since these do not include specific vaccine delivery costing methodologies.

Table 2 shows a comparison of costing term definitions among the various guidance documents. It shows that among the different guidance documents, definitions are generally similar but have differences in wording, e.g., vaccine delivery cost, economic cost, start-up/ introduction cost, and prospective cost. Also, some terms (retrospective costing, cost projections, bottom-up and top-down costing) are only defined in the Global Health Costing Consortium reference case. Note that some guidance documents have been grouped together since they were developed by the same teams; i.e., (i) EPIC documents and (ii) WHO vaccine-specific costing tool user manuals.

**Table 2:** Definitions of costing terms by guidance document

	EPIC (including ‘How to Cost Immunization Programs’ and the Common Approach)	GHCC	ICAN	WHO 2002 Guidelines for Introducing New Vaccines	Costing Tools’ User Manuals (CHOLTOOL, C4P, SIICT)^a^	WHO cMYP Guideline
**Vaccine delivery cost**	All resources used, whether immunization-specific, or ‘shared, and whether consumed at immunization delivery “sites” or above the level of service delivery, with and without the new vaccine (How to cost immunization programs, pg. 4) [12]	NA	Costs associated with delivering immunizations to target populations, exclusive of vaccine costs (pg.11) [6]	NA	Vaccine delivery includes startup costs, service delivery (personnel time, supplies and transport/allowance), vaccine procurement, monitoring and supervision, and other costs (C4P guide, pg. 262) [15] (Not included in other tool manuals)	NA
**Financial cost**	A financial costing is concerned with accounting transactions (i.e., monetary outlays or expenditures) (How to cost immunization programs, pg. 7) [12]	Capture the resources that are “paid” for (pg. A-8) [8]	Financial outlays, usually with straight-line depreciation of capital items (pg. 31) [6]	Actual expenditure for resources used for goods or services purchased. Does not include cost of existing health personnel time or donated goods (pg. 2) [14]	Actual monetary flows of the buyer such as the Ministry of Health. Does not include the value of resources already paid for, such as personnel time (SIICT guide, pg. 21) [17]	NA
**Economic cost**	An economic costing values resources based on their opportunity cost, regardless of whether a financial transaction occurred (How to cost immunization programs, pg. 7) [12]	The value of the highest alternative health intervention opportunity forgone; captures the full value forgone of all resources used (pg. A-8) [8]	Financial outlays plus opportunity costs such as health worker time and any donated items such as vaccines (pg. 56) [6]	Resources that have been foregone for alternative uses, or opportunity costs (pg. 2) [14]	Estimates all costs of an intervention, regardless of the source of funding, so that the opportunity cost of all resources is accounted for in the analysis, includes in-kind and donor contributions (SIICT guide, pg. 21) [17]	NA
Undepreciated financial costs (sometimes called initial investment in costing tool guides and referred to as fiscal costs in previous analyses)	Reflect what governments and donors have paid for activities, services, and goods (Common Approach, pg. 19) [11]	NA	Financial outlays, usually without depreciation of capital items (pg. 31) [6]	NA	Initial upfront resource requirements (C4P guide, pg. 268) [15]	NA
**Recurrent cost**	Recurrent items include labor and consumable items such as vaccines doses, supplies and travel costs (How to cost immunization, pg. 11) [12]	Value of resources/inputs with useful lives of less than one year (pg. 61) [8]	NA	Items that are used up during a year (pg. 3) [14]	Goods or items used in the delivery of a service or intervention that last less than a year, e.g., personnel salaries (SIICT guide, pg. 21) [17] (Not included in all tool manuals)	Costs of resources consumed within one year (CMYP guide, pg. 19) [16]
**Capital cost (sometimes called investment cost)**	Capital items are durable items such as building, equipment, and vehicles (How to cost immunization, pg. 11) [12]	One-time costs for items that have a useful life of over one year (pg. B-23) [8]	NA	Items that last longer than one year and are therefore incurred only every few years rather than annually (pg. 3) [14]	Goods that last for longer than one year, such as equipment (SIICT guide, pg. 21) [17]	An input that has a useful life of more than one year (cMYP guide, pg. 19) [16]
**Incremental cost**	Make assumptions about what particular resources were affected by the intervention, and only measure those resources (How to cost immunization, pg. 8) [12]	Cost of adding a new or a batch of services or intervention over and above an existing program (pg. 59) [8]	Additional costs associated with introducing new vaccines or making changes in delivery (pg. 32) [6]	Only looks at the cost of an addition, e.g., a new vaccine, to existing services (pg. 2) [14]	Additional resources required to add an intervention to an existing immunization program (CHOLTOOL guide, pg. 6) [13] (Not included in other tool manuals)	NA
**Full cost**	Full costs include baseline cost as well as the additional cost of the new intervention (How to cost immunization, pg. 8) [12]	NA	The sum of all costs associated with vaccination delivery (pg. 31) [6]	NA	NA	NA
**Cost projections**	NA	NA	NA	NA	NA	Total future costs of both recurrent and capital inputs to the NIP (cMYP guide, pg. 108) [16]
**Prospective data collection**	Direct observation (How to Cost Immunization Programs, pg. 21) [12]	Direct observation of resource use (pg. B-18) [8]	NA	NA	NA	NA
**Retrospective data collection**	NA	Data collection takes place after resource use (pg. B-18) [8]	NA	NA	NA	NA
**Start-up or introduction costs**	Costs that are incremental to the routine immunization system and specifically incurred as a result of introduction of the new vaccine (Common Approach, pg.6) [11] All resources used for one-time activities (e.g., social mobilization, cold chain capacity mobilization expansion) in a defined time period around the introduction (How to Cost Immunization, pg. 4) [12]	NA	NA	NA	Initial one-time programmatic activities and include micro-planning, initial training activities, and initial sensitization/social mobilization/IEC (SIICT guide, pg.21) [17] (Not included in other tool manuals)	NA

^a^ Similar definitions were included in other tool user manuals unless otherwise noted; NA, not available. Note: This table does not include WHO 1994 from the Consensus Statement since its definitions were not specific to immunization

**Fig. 3:**
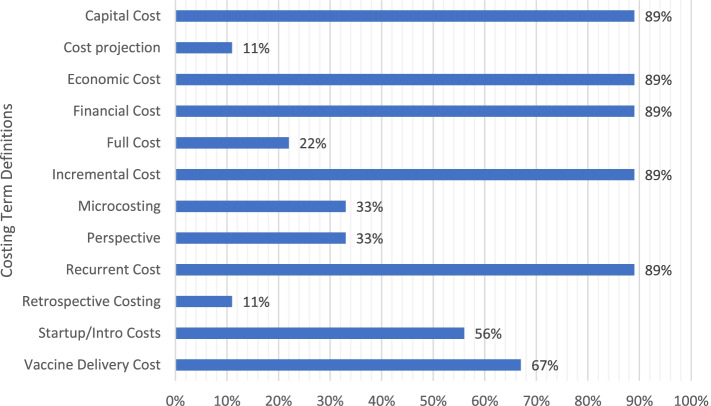
Percentage of guidance documents with definitions of costing terms (*N* = 9)

Figure 3 shows the percentage of guidance documents with definitions of individual costing terms. As can be seen, most documents had definitions of financial cost, economic cost, capital cost, recurrent cost, incremental cost, and vaccine delivery cost, and about half of these defined start-up/introduction cost. Fewer than half of the guidance documents had definitions of perspective, micro-costing (ingredients costing), full costing, retrospective costing, or cost projection.

Several gaps were noted from the review. Most guidance documents did not go into detail about some methodological decision points in costing, such as how the choice of perspective will affect which costs are included as financial costs, which may limit the comparability of such costs across studies. For example, if a payer or provider perspective is used, the organizations included in the study definition as “payers” or “providers” will determine whose monetary outlays are considered as financial costs. If a donor (e.g., Gavi) provides funding to a UNICEF country office for social mobilization for a new vaccine introduction, expenditures of those funds will be included as financial costs only if the study perspective is defined as including UNICEF (e.g., a provider perspective defined as all partners “providing” the new vaccine introduction activities, or a health sector perspective including all health sector partners); however, if the study is conducted from a perspective that does not include UNICEF (e.g., a provider perspective defined as only the government “providing” the new vaccine introduction activities, or a government perspective), these resources from UNICEF would not be counted as financial costs but only as economic costs as an in-kind contribution from UNICEF.

Also, most guidance documents did not address whether to include economic costs of existing capital such as equipment or building space, or how to make or assess assumptions for slackness (i.e., available unused capacity) of existing capital goods. Also, vaccine delivery costing definitions differ on whether actual vaccine product costs should be included or not. If not, which specific aspects of the vaccine product costs should be excluded (e.g., vaccine only, diluent, syringes, safety boxes, freight, and insurance). For financial costs, the guidance review suggested whether to include existing personnel costs will depend on whether the costing is incremental or full.

**Fig. 4:**
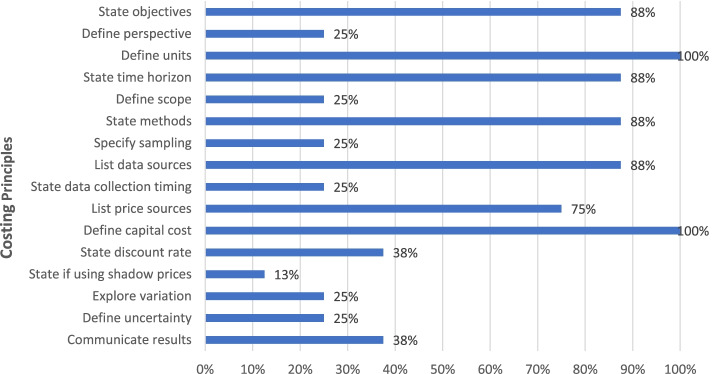
Percentage of costing principles recommended by guidance documents (*N* = 9)

Figure 4 shows the percentage of documents that recommended key costing principles (details in Additional file 1: Table A3). As can be seen, most guidance documents recommended principles on stating objectives, defining units, describing time horizon, methods and data sources, and annualizing capital costs, while less than half recommend specifying the perspective, scope, sampling, data collection timing, discount rates, shadow prices, exploring variation, analyzing uncertainty, and methods of communicating results.

In Table 3, the recommendations of guidance documents on data collection and analysis are disaggregated by workstream. While guidance is given on some aspects in all documents, in other cases, no guidance is provided. Specifically, guidance is given on data collection for all of the workstreams with the exception of projection of new vaccine introduction costs.

**Table 3:** Guidance on data collection and analysis by workstream

	Perspectives included in guidance documents	Data Sources recommended in guidance documents	Data Collection guidance provided	Sampling guidance provided	How is uncertainty characterized?	Source Documents
Retrospective routine immunization cross-sectional costing	Provider, Payer, or Societal	Health facility records; interviews with national and sub-national program managers	Strategies for data collection provided	Representative sampling of health facilities (stratified, random)	Characterized based on number of sites in sample, stratification of units, and basis of probability of selection; one-way sensitivity testing or scenario analysis	[6, 11, 12]
Retrospective single-vaccine costing	Provider payer, or Societal	Interviews with national and sub-national program managers; not described for costing tools	Some advice but not available for costing tools	Representative sampling of health facilities or campaign sites; not specified for costing tools	Characterized based on number of sites in sample, stratification of units, and basis of probability of selection; costing tools are not specific but suggest use of scenarios	[12, 14, 15]
Projection of new vaccine introduction costs	Provider, payer, or Societal	Not described in guidance documents	Provided for some tools but not others	Not specified	Costing tools are not specific but suggest use of scenarios	[11, 13–15, 17]
Projection of Immunization program costs	Provider	Interviews with national and sub-national program managers; visits to selected health facilities sometimes	Provides data collection guidance	Can collect data at the sub-national as well as national levels	Conduct scenario analysis to have a range of estimates	[16]

### Recommended costing terminology and principles

After reviewing the definitions of costing terms, the following definitions of costing terms are recommended:

1. Vaccine delivery costs
  Costs associated with delivering immunization programs to target populations, exclusive of vaccine costs.
2. Vaccine cost
  At a minimum includes the cost of the vaccine and diluent (if applicable); the analysis should include accounting for wastage rates; the analyst should specify whether this also includes injection supplies (syringes), international shipment, insurance, and customs/duties.
3. Financial cost
  Monetary outlays, with straight-line depreciation for capital goods; does not include opportunity costs for use of resources or donated goods and services from sources other than the payer(s) defined in the analysis. Definition is dependent on perspective since monetary outlays are specific to the payer(s) defined in the analysis.
4. Economic cost
  The value of all resources utilized, regardless of the source of financing. Includes opportunity costs for use of existing resources and any donated goods or services from any source. Capital costs are annualized and discounted.
5. Undepreciated financial cost
  Financial costs without depreciation of capital costs (note: such costs have been termed “initial investment” in some costing tools and referred to as fiscal costs in previous analyses.)
6. Recurrent cost
  Value of resources that last less than one year. Start-up activity costs may include recurrent costs.
7. Capital cost
  Value of resources lasting more than one year such as equipment, buildings, and trainings. Start-up activity costs may include capital costs.
8. Incremental cost
  Cost of adding a new service/intervention or a package of services/interventions over and above an existing program; inclusion of existing resources will depend on assumptions made about excess capacity (i.e., whether resources are underemployed; if there are no slack resources (e.g., all personnel time is fully allocated before the addition of the new service/intervention), then their use for the new service or intervention incurs an opportunity cost that should be included—either by measurement or assumption).
9. Full cost
  Baseline cost as well as the additional/incremental cost of the new intervention, including vaccine cost.
10. Cost projection
  Estimation of future costs of both recurrent and capital inputs.
11. Prospective data collection
  Direct observation of resource use during intervention implementation; i.e., data are collected concurrently with intervention implementation.
12. Retrospective data collection
  Data collection after resource use is completed.
13. Start-up cost
  Cost of initial one-time programmatic activities. Examples may include initial micro-planning, initial training activities, and initial sensitization/social mobilization/information, education, and communication (IEC); does not include routine or repeated programmatic activities such as refresher training or annual microplanning. Start-up activities may include both recurrent and capital costs; they are defined by the non-repeating nature of the activity, not the type of input.
14. Micro-costing
  Focuses on granular accounting of input prices and quantities; disaggregates costs of particular output into specific goods and services consumed.
15. Bottom-up costing
  Measures input quantities at the client (e.g., per vaccination administered) or activity level.
16. Top-down costing
  Divides overall program cost or expenditures, often including those at administrative levels above service delivery level, by number of outputs to calculate unit cost.
17. Perspective
  The point of view considered for costs (and benefits, if included) in a costing study, by whom the costs were incurred. Payers are the disbursing agents for a good or service, and may differ from the original source of funding. A provider perspective includes costs incurred by health service providers (can be limited to the government), a payer perspective includes costs to the payer(s), such as government or an external partner, while the societal perspective includes all costs incurred by providers as well as clients.
18. Shared cost
  Shared resources that are not used only for immunization, but also for other productive activities.

The recommended costing principles include the following.

1. Definitions of terms used in studies of vaccine delivery costing should conform closely to the recommended definitions in this Consensus Statement.
2. The study scope in terms of its purpose, audience, target population, time horizon, and service/output should be clearly stated. It should also state whether data collection will be prospective or retrospective, and whether the analysis will be retrospective or a cost projection.
3. The perspective of the cost estimation should be stated and justified.
4. Types of costs to be generated should be clearly defined in terms of start-up/introduction or non-start-up/introduction (sometimes called operating costs), recurrent and capital, undepreciated financial, financial or economic, and incremental or full. Capital costs should be appropriately annualized and depreciated for financial and economic costs and the discount rate justified.
5. The scope of the inputs to be estimated should be defined, justified, and if needed referenced. For example, do the costs include national and sub-national costs or only facility-level service delivery costs? Are non-immunization costs included?
6. The “units” in the unit costs for strategies, services, and interventions should be defined, e.g., cost per dose administered.
7. If incremental costing is conducted, any assumptions made regarding existing health system capacity should be described (see GHCC reference case, pg. 64).
8. The selection of the data sources, including any adjustments to price data (e.g., inflation or currency conversion) should be described and referenced.
9. The methods for estimating the quantity of inputs should be described—whether top-down or bottom-up, methods of allocation, use of shadow prices and the opportunity cost of time, and methods for excluding research and evaluation costs.
10. Costs should be mapped and reported as either inputs or activities:
  i. Resource inputs include, for example, personnel time, vaccines, injection and safety supplies, vehicles, fuel, per diem and travel allowances, cold chain equipment, stationery, laboratory equipment, and buildings;
  ii. Program activities include, for example, vaccine procurement, service delivery, training, micro-planning, social mobilization, and advocacy and communication, monitoring and evaluation, surveillance, adverse event following immunization monitoring, and supervision.
11. Some boundaries around costs included in the analysis may be employed to keep the costing scope feasible and will depend on the purpose of the costing study, with the rationale for any exclusions provided; use discretion about including one-time costs that are unique or unlikely to be replicated or transferable across settings (for example, new vaccine launches with the President). Clarify definition and threshold for including or excluding small costs that have expected small contribution (e.g., <$25) to total costs in aggregate across all sampled units, such as the use of existing office supplies by health facility staff.
12. The sampling strategy employed should aim for internal and external validity of the data^3^. Sampling strategy should be stated, described, and justified, depending on the workstream and costing objectives. Sampling of different service delivery units is desirable as it provides a more representative picture of costs and highlights cost variation and cost drivers for a strategy or vaccine.
13. Variation in the cost of the intervention by site/organization, sub-population, or by other drivers of heterogeneity should be explored and reported for retrospective analyses when possible.
14. The uncertainty around the cost estimates should be appropriately characterized when feasible, (e.g., sensitivity analyses; ranges of results for different input parameter scenarios for cost projections; mean and standard deviation for non-representative samples with multiple units; and confidence intervals or credible intervals for retrospective analyses).
15. Inclusion and exclusion criteria: “stopping rules”^4^ should be defined, explaining which costs are included and the respective rationale.
16. Cost estimates should be communicated clearly and transparently to enable decision-makers to interpret and use the results relevant to the original policy and/or programmatic question.

## Discussion

The lack of standardization in terminology, implementation, and principles for vaccine delivery costing has resulted in difficulties in making comparisons among studies, reducing the potential for synthesis of economic evidence across studies for immunization program policy, planning, budgeting, and implementation. As noted earlier, governments need to know the cost of vaccine delivery in order to make decisions on introducing new infant and life-course vaccines, budgeting, and for making improvements in service delivery. The review indicates that existing guidance documents differ somewhat in the inclusion and definitions, of costing terms and costing principles that are recommended, reflecting in part differences in the aims and scope of the costing study.

The review of guidance documents and tools on vaccine delivery costing and iterative discussions among the Working Group members revealed considerable agreement among the different groups working in vaccine delivery costing. Most of the documents made the distinction between economic and financial costs as well as recurrent and capital costs. However, fewer went into detail about the perspective to choose, definition of some costing terms such as start-up costs, micro-costing, and bottom-up/top-down costing, and in some cases, recommended approaches for data collection and analyses. The review also identified gaps in guidance for some analyses, e.g., such as how perspective affects financial costs calculation.

The review revealed that different workstreams focus on distinct aspects of immunization costing with different purposes. These require different types of data collection and analyses. For example, retrospective costing of vaccination focuses on estimating actual resource use, benchmarking of costs, and investigation of variation at the facility and other levels. Cost projections, on the other hand, focus on estimation of (typically incremental) costs to assist in decision-making, preparation of budgets, and evaluating different approaches to a new technology, vaccine, or service delivery strategy.

The process to achieve a consensus statement of vaccine delivery costing methods was facilitated by having extensive consultations with different organizations conducting this work. It also was facilitated by conducting reviews of the guidance documents and costing tools so that similarities, differences, and gaps could be identified. Other strengths of the process include broad and ongoing engagement of experts across various workstreams, including members of the Immunization Economics Community of Practice [18], as well as dedicated support for facilitation, review, and write-up.

The process to develop a consensus statement provides lessons for developing agreement among other organizations and researchers on types of research methods and tools in other study areas. It requires the potential to bring together organizations working on similar research and then having the time and resources to develop consensus. In addition, it is useful to have some teleconferences and in-person (or virtual meetings with break-out sessions) meetings to have sufficient time to come to consensus.

One limitation of the exercise was that a systematic review was not conducted and some guidelines and costing tools may have been missed. More engagement of country-level practitioners and data and analysis experts outside of those directly involved in the workstreams, as well as a systematic literature search for any methodology documents beyond those known to the workstream participants, would have strengthened the process.

The work on immunization costing is extensive but some gaps were identified. The guidance documents, mostly user manuals for costing tools and the 2002 WHO guidance on introducing new vaccines for cost projections of new vaccines, are not sufficiently detailed regarding data collection and analyses. That is, these do not include instructions on methods of data collection and sampling and analysis methods, when required. Researchers that have piloted the costing tools have also noted that the manuals need to provide more instructions on perspective (see [19], for example). For example, there is a need for more guidance on how to treat perspective when there is more than one source of financing of vaccines, how to handle slack, etc. As a result, it would be useful to add to current user manuals or develop a new guidance document for cost projections for both single vaccines, multiple vaccines, and immunization programs.

## Conclusions

This review and Consensus Statement development process revealed the limitations of our ability to harmonize given that study designs will vary depending upon the policy question that is being addressed and the country context. The Working Group hopes that the consensus statement will contribute to the development of costing guidelines and tools for new vaccines (single or multiple) and immunization programs that are better aligned in terms of definitions, methods, and reporting.

## Supplementary Information

**Additional file 1. **Consensus Statement on Vaccine Delivery Costs which includes a review of vaccine delivery cost guidance documents and costing tools as well as a consensus statement on the terminology and methodological principles to be used for vaccine delivery costing. It includes two figures and 6 tables. **Figure S1.** Major current workstreams in vaccine delivery costing identified by working group. **Figure A1** – Timeline for developing a Consensus Statement on Vaccine Delivery Costs. **Table A2a.** List of guidelines by publication year, target interventions, and purposes. **Table A2b.** List of costing tools for vaccine delivery or immunization program. **Table A3.** Definitions of costing terms in guidance documents. **Table A4.** Comparison of costing principles among guidance. **Table A5.** Characteristics of costing workstreams. **Table 6A.** Data sources, sampling and characterization of uncertainty, and terminology by workstreams.

## Data Availability

All data generated or analyzed during this study are included in this published article and its additional file.

## Abbreviations

2YL: 2nd Year of Life
BMGF: Bill & Melinda Gates Foundation
C4P: Cervical Cancer Prevention and Control Costing
CDC: United States Centers for Disease Control and Prevention
CHOLTOOL: Oral Cholera Vaccine Costing Tool
cMYP: Comprehensive multi-year plan
CS: Consensus statement
EPIC: Expanded Programme on Immunization Costing
FIP: Fully immunized person
GHCC: Global Health Cost Consortium
ICAN: Immunization Costing Action Network
IEC: Information, education, and communication
iHEA: International Health Economics Association
IVI: International Vaccine Institute
IVIR-AC: Immunization and Vaccines-related Implementation Research Advisory Committee
LIC: Low-income country
MIC: Middle-income country
MVICT: Malaria Vaccine Immunization Costing Tool
SIICT: Seasonal Influenza Immunization Costing Tool
TCVCT: Typhoid Conjugate Vaccine Costing Tool
VTIA: Vaccine Technology Costs and Health Impact Assessment Tool
WHO: World Health Organization

## Footnotes

1 The experts in the Working Group were from the World Health Organization, UNICEF, US Centers for Disease Control and Prevention, the Bill & Melinda Gates Foundation, Harvard T.H. Chan School of Public Health, International Vaccine Institute, ThinkWell, and the London School of Hygiene and Tropical Medicine.

2 Two presentations were made: (1) the WHO team’s Ann Levin presented on WHO/IVI/PATH’s work conducting vaccine delivery cost projections with costing tools and the lack of standardization with other workstreams; and (2) ThinkWell’s Annette Ozaltin, representing the BMGF portfolio, presented their work on vaccine costing and the repository of vaccine delivery costs known as the Vaccine Cost Catalogue.

3 Internal validity refers to the extent of systematic bias in an estimate while external validity is the extent to which the cost estimate can be directly applied to other programmatic setting. (GHCC, pg. A15–A16).

4 A “stopping rule” defines and explains which costs are included, and how the line is drawn between inclusions and exclusions. (GHCC reference case, pg. B-2)
