# Supplementary material for: WHO-led consensus statement on vaccine delivery costing: process, methods, and findings
Source: BMC Med. 2022 Mar 8;20:88. doi: 10.1186/s12916-022-02278-4 (PMC8902809; doi:10.1186/s12916-022-02278-4)
Supplement: Supplementary file 1 — Additional file 1. Consensus Statement on Vaccine Delivery Costs which includes a review of vaccine delivery cost guidance documents and costing tools as well as a consensus statement on the terminology and methodological principles to be used for vaccine delivery costing. It includes two figures and 6 tables. Figure S1. Major current workstreams in vaccine delivery costing identified by working group. Figure A1 – Timeline for developing a Consensus Statement on Vaccine Delivery Costs. Table A2a. List of guidelines by publication year, target interventions, and purposes. Table A2b. List of costing tools for vaccine delivery or immunization program. Table A3. Definitions of costing terms in guidance documents. Table A4. Comparison of costing principles among guidance. Table A5. Characteristics of costing workstreams. Table 6A. Data sources, sampling and characterization of uncertainty, and terminology by workstreams. [file 12916_2022_2278_MOESM1_ESM.pdf]

Consensus Statement on Vaccine Delivery Costs

April 2021

Authors

Ann Levin, Levin & Morgan LLC

Laura Boonstoppel ^, ThinkWell

Logan Brenzel ^, Bill & Melinda Gates Foundation

Ulla Griffiths ^, UNICEF

Xiao Xian Huang ^, World Health Organization

Raymond Hutubessy ^, World Health Organization

Mark Jit ^, London School of Hygiene & Tropical Medicine

Vittal Mogasale ^, International Vaccine Institute

Sarah Pallas ^, Centers for Disease Control and Prevention

Stephen Resch ^, Harvard T.H. Chan School of Public Health

Christian Suharlim ^, Harvard T.H. Chan School of Public Health

Stéphane Verguet ^, Harvard T.H. Chan School of Public Health

Karene Hoi Ting Yeung ^, World Health Organization

^ Authors contributed equally and listed in alphabetical order

Disclaimer: The views in this work are those of the authors in their individual capacities and do not represent the official positions of the authors' organizations.

1. Background

This consensus statement was developed in response to a request from the World Health Organization (WHO) Immunization and Vaccines related Implementation Research Advisory Committee (IVIR-AC). They requested that 'guidance be developed for standardization of micro-costing and planning tools, on new vaccine introduction on where to obtain data, at what level it should be collected, how to conduct sampling, and methods used in vaccine delivery costing.' (Weekly Epidemiological Record, No. 24, 15 June 2018). In response to this request, an *ad hoc* Working Group was created to oversee the development of this guidance for WHO.

The Working Group identified that multiple efforts, either in process or completed, each partly addressed or are addressing the original IVIR-AC request on data collection, sampling, and methods to be used in vaccine delivery costing. Each of these efforts has different purposes. With this new information, in March 2019, IVIR-AC modified their request to instead review and document the various workstreams that are being conducted in immunization costing. In July 2019, the Working Group met to discuss the vaccine delivery costing work taking place through different organizations. They noted some differences in terminology and principles among the organizations. As a result, they agreed to develop this consensus statement to harmonize key terminology and clarify the scope of the various methods. Annex 1 shows a figure that illustrates the chronology of presentations to IVIR-AC on vaccine delivery costing and other meetings to develop the Consensus Statement.

The target audience for the consensus statement is the developers of costing tools or guidance, vaccine delivery cost researchers, and funders of costing tools, guidance and studies. The expectation is that the terminology and methods utilized in the future for developing new tools or guidance, undertaking delivery cost studies, interpreting findings on vaccine delivery costs, or reviewing studies/research/tools will be consistent with this Consensus Statement. It recognizes that retroactive changes to published costing tools and guidance documents that differ from the recommended terminology and methods may not be feasible. The Consensus Statement summarizes similarities and differences in data collection and sampling methods among costing approaches as well as gaps in guidance documents.

2. Objectives of the Consensus Statement for the Immunization Costing Community

The objectives of the consensus statement are the following:

- To highlight and explain commonalities and differences across different costing approaches, tools, and guidelines;
- To highlight the objectives of different costing approaches, tools, and guidelines;
- To encourage improvement and innovation in methods and tools that are fit for purpose;
- To advance the immunization economics community of practice by committing to follow certain principles and common definitions (as detailed in Annex) that will make the collective costing work more easily interpretable and useful, while acknowledging that some deviations may occur due to limits to standardization of approaches with different objectives.

DRAFT

To achieve these objectives, the Working Group reviewed terminology, definitions, and principles of guidance documents and costing tools for vaccination delivery. Recommendations for costing principles and terminology were developed.

For the purpose of this document, the definition of vaccine delivery costing is the following: costs associated with delivering immunizations to target populations, exclusive of vaccine costs. Vaccine delivery costs can be disaggregated into financial and economic delivery costs (see cost definitions below).

3. Vaccination Delivery Cost Analyses

Efforts to estimate the costs of immunization programs, strategies, and new vaccine introductions have utilized various methodological approaches as described below. The approach selected is usually based on the purpose of the analysis and the type of information that decision-makers need. The Working Group qualitatively characterized workstreams based on their knowledge of groups currently working in the field following a 2019 International Health Economics Association pre-congress session on vaccine economics in Basel, Switzerland. These characterizations were intended to help elucidate where and why differences in definitions and methods were occurring and were not derived from any prior framework.

Major workstreams on costing of vaccine delivery and immunization program costing identified by the Working Group are the following:

i. **Retrospective routine immunization (multiple vaccines) cross-sectional costs:** The first workstream is focused on estimating retrospective (i.e., already incurred) routine immunization, cross-sectional costs of service delivery units at a single point in time, typically using a full costing approach. This method provides a range of unit costs (cost per dose, cost per person, cost per fully immunized person [FIP]) by facility, district, and higher levels in the health system for total routine immunization delivery costs. Costs are economic and/or financial costs. It includes, for example, the work conducted in the Expanded Programme on Immunization Costing (EPIC) studies (see www.immunizationeconomics.org) and other work by groups, such as the Harvard School of Public Health, Wits University, Curatio Foundation, PAHO, ThinkWell, UNICEF, Johns Hopkins University, and PATH (see Annex 2 for details). The purposes are to determine delivery costs of the entire routine immunization program as it currently operates for benchmarking and/or to explain variation in facility costs and unit costs (e.g., cost determinants, efficiency).

II. **Retrospective single-vaccine costs:** The second approach is to estimate retrospective costs for a specific vaccine, typically using incremental costing. Retrospective estimation of incremental vaccine-specific campaign and new vaccine introduction costs differs from full costing of routine immunization in requiring some implicit or explicit estimation of counterfactual resource use in the absence of that campaign or vaccine introduction. This is often done through data collection at a single point in time (post-campaign or post-introduction) with reference to documents and recall by key informants to estimate which

resource use was specifically incremental. Examples of such studies are being applied by groups such as ThinkWell, Harvard School of Public Health (EPIC studies), International Vaccine Institute (IVI), WHO, UNICEF and Centers for Disease Control and Prevention (CDC). Costing tools used to estimate retrospective costs include, but are not limited to (see Annexes for websites for these tools):

- the IVI/WHO CHOLTOOL
- the WHO Cervical Cancer Prevention and Control Costing Tool (C4P)
- the WHO Seasonal Influenza Immunization Costing Tool (SIICT)
- the WHO/IVI Typhoid Vaccine Costing Tool (TCVCT)
- the PATH Malaria Vaccine Immunization Costing Tool (MVICT)
- the PAHO ProVac/COSTVAC

Incremental costing of a specific vaccine, whether delivered through campaign or routine, differs substantially from full costing of routine immunization because it involves not only estimating the proportion of shared health system resources used for immunization, but also the extra step of allocation by vaccine. In particular, campaign delivery may differ in frequency, administrative levels (sometimes sub-national rather than national), whether these are preventive or in response to outbreaks (e.g., oral cholera vaccine [OCV] provision), for catch-up, and whether these involve populations other than young children and pregnant women, such as health workers, adolescent girls, or all ages over one year for OCV. When conducted for a campaign, the purpose of these cost analyses may be for retrospective evaluation of campaign costs (including as an input to cost-effectiveness analyses), explaining variation in costs by strategy and venue, and cost projections for planning and decision-making on conducting campaigns. When estimating retrospective costs of new vaccine introduction, whether via campaign or routine immunization, the purpose of these analyses may be to inform country planners and decision makers, and global funders on the costs of introduction and recurrent costs over time. Both financial and economic costs are estimated.

iii. **Projection of new vaccine introduction costs:** The third approach is estimation of new vaccine introduction costs through the projection of the price and quantity of ingredients (e.g., time, equipment, vaccines, etc.) needed for vaccine introduction, typically using incremental costing for a specific period, e.g., one or five years. The prices and quantities of ingredients are obtained through interviews with program managers and facility visits to obtain current information on for instance personnel time, supplies, and equipment. The projections are often conducted with the same costing tools as found in the second workstream: C4P, SIICT, TCVCT, CHOLTOOL, and MVICT. Examples of such studies have been funded by BMGF through EPIC, WHO, UNICEF, IVI, CDC, and PATH. Another tool, the Vaccine Technology Costs and Health Impact Assessment Tool (VTIA), is used to compare the commodity and system costs for a new vaccine technology (e.g. temperature stability vaccines) with the current one. The purpose of these cost projections is for planning and decision-making on new vaccines during the introduction period. Costs are shown for both financial and economic costs and include cost per dose and FIC as well as total annual costs.

DRAFT

iv. **Projection of national immunization program costs**: The fourth workstream is immunization program cost projection (e.g., comprehensive multi-year plan [cMYP], 2nd Year of Life (2YL), OneHealth tool) where the activities of a national program and related cost is approximated for a baseline year and then the costs of future years are projected. This is a type of costing for strategic planning to assist in budgeting, resource planning, and mobilization over a strategic period. These projections estimate undepreciated financial costs; also, both annual and three to five-year costs are estimated.

Figure S1 shows the four workstreams, their lead agencies/funders, and associated guidance documents/tools.

Figure S1. Major Current Workstreams in Vaccine Delivery Costing identified by Working Group

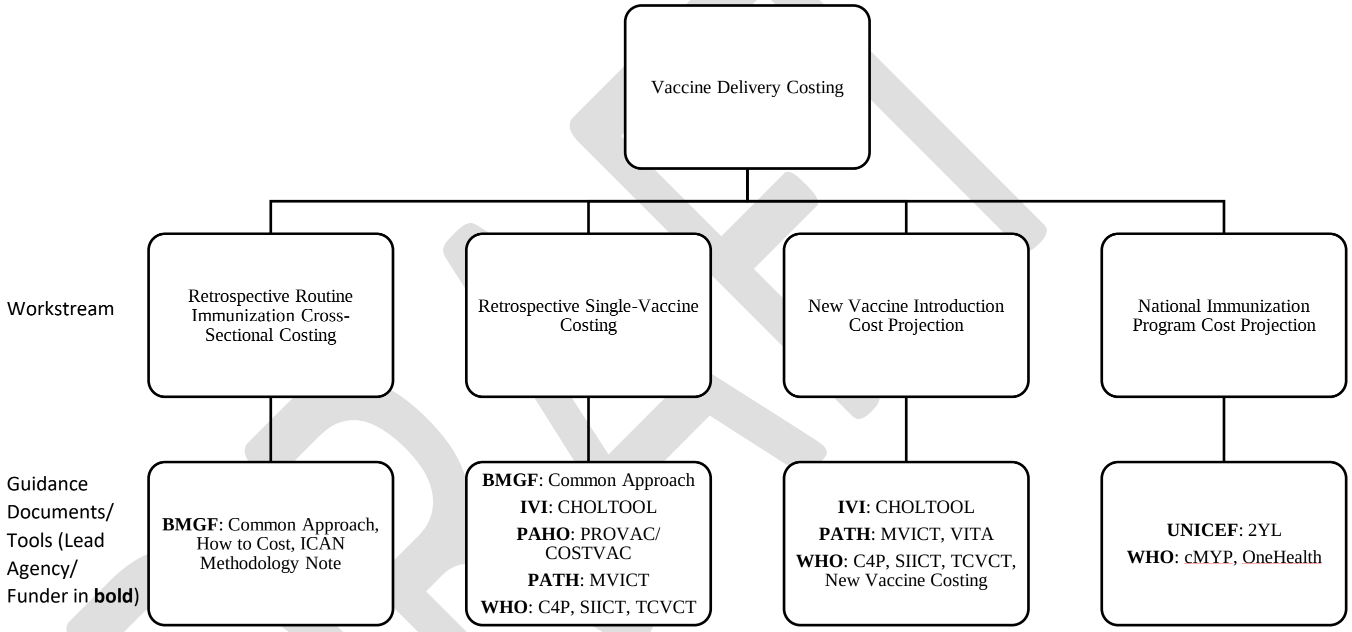

Note: 2YL = 2nd Year of Life; BMGF = Bill & Melinda Gates Foundation; C4P = Cervical Cancer Prevention and Control Costing; CDC = United States Centers for Disease Control and Prevention; CHOLTOOL = Oral Cholera Vaccine Costing Tool; cMYP = comprehensive multi-year plan; EPIC = Expanded Programme on Immunization Costing; ICAN = Immunization Costing Action Network; IVI = International Vaccine Institute; MVICT = Malaria Vaccine Immunization Costing Tool; SIICT = Seasonal Influenza Immunization Costing Tool; TCVCT = Typhoid Conjugate Vaccine Costing Tool; VTIA = Vaccine Technology Costs and Health Impact Assessment Tool; WHO = World Health Organization

DRAFT

4. Review of existing Guidance Documents and Costing Tools

Annex Tables A2a and A2b show the eleven existing guidance documents and eleven tools for costing vaccine delivery and immunization programs. These were identified by the Working Group as of July 2020. Note that this list is based on Working Group members' personal knowledge and prior reference to them in conducting immunization delivery costing and may not be exhaustive. A few of these guidance documents and tools are for costing health services more generally, such as the OneHealth Tool and the Community Health Planning and Costing Tool (CHPCT).

The review showed that some gaps in costing guidance for the workstreams exist on how to consider slackness of resources, estimation of shared resources for the interventions and specific vaccines, and sampling and respondent selection, particularly for the cost projections for vaccine introduction.

Terminology and definitions of costs in workstreams

Annex Table A3 shows definitions of costing terminology found in the guidance documents. The guidance documents have similar definitions of financial and economic costs, and recurrent and capital costs, but vary in the level of details of the definitions. Most guidance documents do not describe in detail issues of interactions [1] between terminology, perspective, financial vs. economic costs [2], and how incremental costing affects financial vs. economic costing [3]. For example, incremental costs for financial costs will differ depending on the perspective of the analysis; if the perspective is of the public health provider, resources donated by external entities will not be included.

Annex Table A4 compares the costing principles in the guidance documents with the Global Health Cost Consortium (GHCC) Principles and Methods Reporting Checklist (GHCC 2017). Guidance documents vary in the extent to which costing principles are discussed. Most guidance documents refer to study purpose, classification of costs, the time horizon of data collection, presentation of costing methods, and depreciation of capital costs. Other principles such as describing the timing of data collection and listing sources for price data are only discussed by one or two of the guidance documents (see discussion in annex).

Annex Table A5 compares the level of data collection, activities/cost categories, perspective, and definitions of cost terms and perspective among the workstreams and shows the variations among these. Annex Table A6 shows differences in data sources, sampling, and characterization of uncertainty by workstream.

[1] An Interaction is the action or influence of things on one another (Merriam-Webster.com).
[2] Financial costs only include resources paid for by the 'buyer' or 'provider' and will therefore be affected by the perspective chosen for the analysis.
[3] The definitions are not clear about whether resources that already exist before the intervention (e.g. cold chain equipment) should be included in economic costs and how excess capacity should affect these (e.g., whether the costs should only be included if there is no slack capacity to absorb the new intervention resource requirements).

Areas for clarification and harmonization

Based on the review, some specific areas that need further clarification and harmonization have been identified in terms of data collection, sampling, and characterization of uncertainty. These are shown in Annex 6.

5. Recommendations for Costing Terms by Working Group

The Working Group reviewed costing term definitions in the existing guidance documents. Based on the definitions shown in Table A2, they developed recommendations for costing terms to be used in estimates of vaccine delivery cost.

The following definitions of costing terms are recommended by the Working Group:

1. **Vaccine delivery costs**
   Costs associated with delivering immunization programs to target populations, exclusive of vaccine costs.
2. **Vaccine cost**
   At a minimum includes the cost of the vaccine and diluent (if applicable); the analysis should include accounting for wastage rates; the analyst should specify whether this also includes injection supplies (syringes), international shipment, insurance, and customs/duties
3. **Financial cost**
   Monetary outlays, with straight-line depreciation for capital goods; does not include opportunity costs for use of resources or donated goods and services from sources other than the payer(s) defined in the analysis. Definition is dependent on perspective since monetary outlays are specific to the payer(s) defined in the analysis.
4. **Economic cost**
   The value of all resources utilized, regardless of the source of financing. Includes opportunity costs for use of existing resources and any donated goods or services from any source. Capital costs are annualized and discounted.
5. **Undepreciated financial cost**
   Financial costs without depreciation of capital costs. (Note: Such costs have been termed "initial investment" in some costing tools and referred to as fiscal costs in previous analyses.)
6. **Recurrent cost**
   Value of resources that last less than one year. Start-up activity costs may include recurrent costs.
7. **Capital cost**
   Value of resources lasting more than one year such as equipment, buildings, and trainings. Start-up activity costs may include capital costs.
8. **Incremental cost**
   Cost of adding a new service/intervention or a package of services/interventions over and above an existing program; inclusion of existing resources will depend on assumptions made about excess capacity (i.e., whether resources are underemployed; if there are no slack resources (e.g., all personnel time is fully allocated before the addition of the new service/intervention), then

DRAFT

their use for the new service or intervention incurs an opportunity cost that should be included – either by measurement or assumption).

9. **Full cost**
   Baseline cost as well as the additional cost of the new intervention, including vaccine cost.
10. **Cost projection**
    Estimation of future costs of both recurrent and capital inputs.
11. **Prospective data collection**
    Direct observation of resource use during intervention implementation, i.e., data are collected concurrently with intervention implementation.
12. **Retrospective data collection**
    Data collection after resource use is completed.
13. **Start-up cost**
    Cost of initial one-time programmatic activities. Examples may include initial micro-planning, initial training activities, and initial sensitization/ social mobilization/ information, education and communication (IEC); does not include routine or repeated programmatic activities such as refresher training or annual microplanning. Start-up activities may include both recurrent and capital costs; they are defined by the non-repeating nature of the activity, not the type of input.
14. **Micro-costing**
    Focuses on granular accounting of input prices and quantities; disaggregates costs of particular output into specific goods and services consumed.
15. **Bottom-up costing**
    Measures input quantities at the client (e.g. per vaccination administered) or activity level.
16. **Top-down costing**
    Divides overall program cost or expenditures, often including those at administrative levels above service level, by number of outputs to calculate unit cost.
17. **Perspective**
    The point of view considered for costs (and benefits, if included) in a costing study, by whom the costs were incurred. Payers are the disbursing agents for a good or service, and may differ from the original source of funding. A provider perspective includes costs incurred by health service providers (can be limited to the government), a payer perspective includes costs to the payer(s), such as government or an external partner, while the societal perspective includes all costs incurred by providers as well as clients.
18. **Shared cost**
    Shared resources that are not used only for immunization, but also for other productive activities.

6. Recommendations for Costing Principles for the Methodological Approaches

The Working Group reviewed costing principles in the various guidance documents and compared these to the GHCC reference case since this document has the most comprehensive set of principles for health service costing. Based on a review of similarities and differences among the guidance documents, they developed recommendations for the costing principles to be used in future costing studies.

DRAFT

The recommended costing principles include the following.

1. Definitions of terms used in studies of vaccine delivery costing should conform closely to the recommended definitions in this Consensus Statement.
2. The study scope in terms of its purpose, audience, target population, time horizon, and service/output should be clearly stated. It should also state whether data collection will be prospective or retrospective, and whether the analysis will be retrospective or a cost projection.
3. The perspective of the cost estimation should be stated and justified.
4. Types of costs to be generated should be clearly defined in terms of startup/introduction or non-startup/introduction (sometimes called operating costs), recurrent and capital, undepreciated financial, financial or economic, and incremental or full. Capital costs should be appropriately annualized and depreciated for financial and economic costs and the discount rate justified.
5. The scope of the inputs to be estimated should be defined, justified and if needed referenced. For example, do the costs include national and sub-national costs or only facility-level service delivery costs? Are non-immunization costs included?
6. The 'units' in the unit costs for strategies, services and interventions should be defined – e.g., cost per dose administered.
7. If incremental costing is conducted, any assumptions made regarding existing health system capacity should be described. (See GHCC reference case, pg. 64).
8. The selection of the data sources, including any adjustments to price data (e.g., inflation or currency conversion) should be described and referenced.
9. The methods for estimating the quantity of inputs should be described – whether top-down or bottom-up, methods of allocation, use of shadow prices and the opportunity cost of time, and, methods for excluding research and evaluation costs.
10. Costs should be mapped and reported as either inputs or activities:
    i. Resource inputs include, for example, personnel time, vaccines, injection and safety supplies, vehicles, fuel, per diem and travel allowances, cold chain equipment, stationery, laboratory equipment, and buildings;
    ii. Program activities include, for example, vaccine procurement, service delivery, training, micro-planning, social mobilization and advocacy and communication, monitoring and evaluation, surveillance, adverse event following immunization (AEFI), monitoring, and supervision.
11. Some boundaries around costs included in the analysis may be employed to keep the costing scope feasible and will depend on the purpose of the costing study, with the rationale for any exclusions provided; use discretion about including one-time costs that are unique or unlikely to be replicated or transferable across settings (for example, new vaccine launches with the President. Clarify definition and threshold for small costs that have expected small (e.g., <$25) contribution to total costs in aggregate across all sampled units, such as the use of existing office supplies by health facility staff.
12. The sampling strategy employed should aim for internal and external validity of the data[4]. Sampling strategy should be stated, described, and justified, depending on the workstream and

DRAFT

[4] Internal validity refers to the extent of systematic bias in an estimate while external validity is the extent to which the cost estimate can be directly applied to other programmatic setting. (GHCC, pg. A15-A16).

costing objectives. Sampling of different service delivery units is desirable as it provides a more representative picture of costs and highlights cost variation and cost drivers for a strategy or vaccine.

13. Variation in the cost of the intervention by site/organization, sub-population, or by other drivers of heterogeneity should be explored and reported for retrospective analyses when possible.
14. The uncertainty around the cost estimates should be appropriately characterized when feasible, (e.g., sensitivity analyses; ranges of results for different input parameter scenarios for cost projections; mean and standard deviation for non-representative samples with multiple units; and confidence intervals or credible intervals for retrospective analyses).
15. Inclusion and exclusion criteria: 'stopping rules[5]' should be defined, explaining which costs are included and the respective rationale.
16. Cost estimates should be communicated clearly and transparently to enable decision-makers to interpret and use the results relevant to the original policy and/or programmatic question.

DRAFT

[5] A 'stopping rule' defines and explains which costs are included, and how the line is drawn between inclusions and exclusions. (GHCC reference case, pg. B-2)

Annexes for the Consensus Statement on Vaccine Delivery Costs (April 2021)

Contents

Annex 1. Timeline for developing a Consensus Statement on Vaccine Delivery Costs (CS) 2
Annex 2. List of existing guidance and costing tools for vaccination delivery costing 3
Annex 3. Definition of costing terminology 7
Annex 4. Costing principles 14
Annex 5. Characteristics of costing workstreams 19
Annex 6. Areas for clarification and harmonization 22

DRAFT

Annex 1. Timeline for developing a Consensus Statement on Vaccine Delivery Costs (CS)

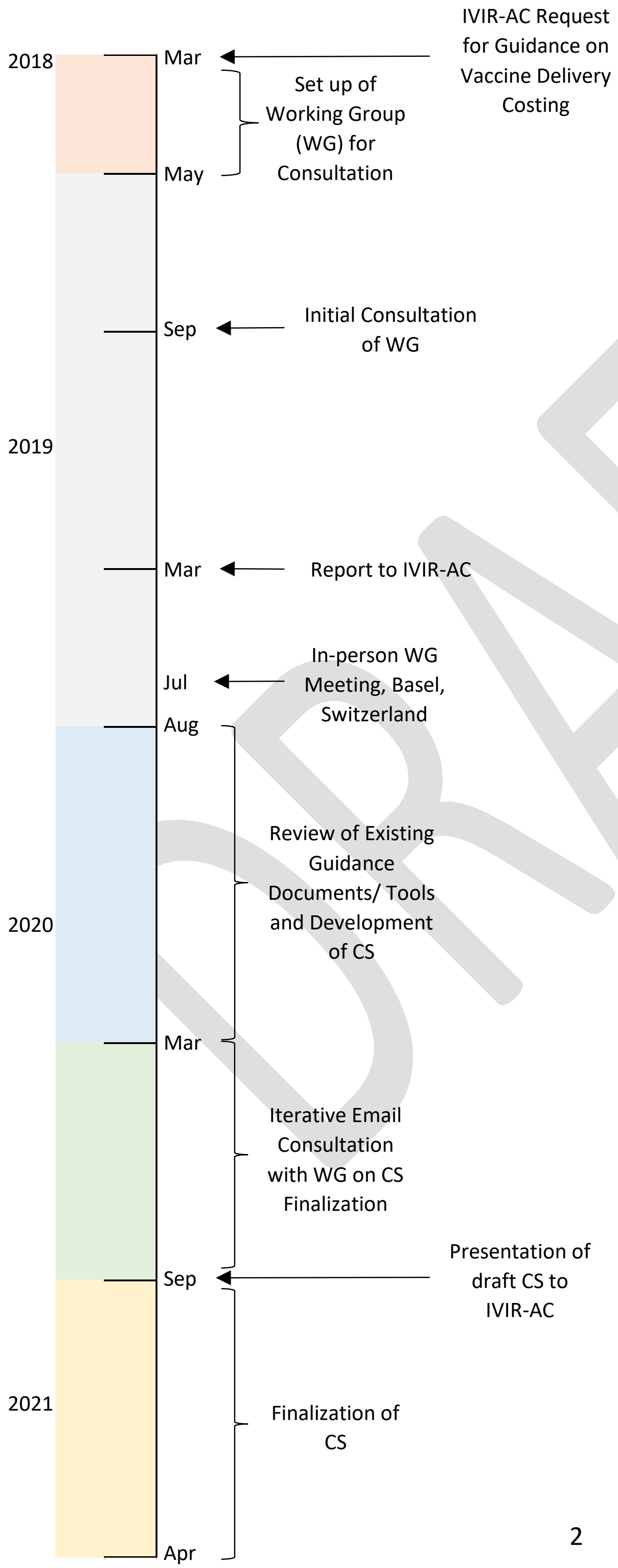

Annex 2. List of existing guidance and costing tools for vaccination delivery costing

Table A2a presents the list of guidance documents with their year of publication, target interventions, and purpose as identified by the advisory group. One document is a training manual for costing primary health care services, one document is a reference case for costing global health care interventions, and the rest are specifically about costing of vaccine delivery. Note that some publications such the textbook on vaccine economics are forthcoming and are not shown in the table.

Table A2a. List of guidelines by publication year, target interventions, and purposes

Developer	Guidelines	Publication years	Target Interventions	Purposes	Link
WHO	Cost analysis in primary health care: A training manual for program managers	1994	Primary health care	Assist health program managers to cost their services for planning and evaluating efficiency	https://apps.who.int/iris/handle/10665/40030
WHO	Guidelines for estimating costs of introducing new vaccines into the national immunization system	2002	New vaccine programs	Assist countries in planning for introduction of new vaccines	https://apps.who.int/iris/handle/10665/67342
	WHO guide for standardization of economic evaluations of immunization programs	2008 (edition I), 2019 (edition II)	Existing and new vaccine programs	Methodology for cost and cost-effectiveness analyses of vaccines	https://apps.who.int/iris/bitstream/handle/10665/69981/WHO_IVB_08.14_eng.pdf;jse https://apps.who.int/iris/rest/bitstreams/retrieve
EPIC	Common Approach for the costing and financing analyses of routine immunization and new vaccine introduction costs	2013	Existing and new vaccine programs	Methods for data collection for routine programs and new vaccine introduction (including delivery costs) and financial flows	http://static1.squarespace.com/static/556deb8ee4b08a534b8360e7/t/55970258e4b03cf942da51ac/1435959896232/WEBSITE_Common+Approach.pdf
	How to Cost Immunization Programs - A practical guide on primary data collection and analysis	2020		Practical guidance on how to conduct a facility-based exercise on immunization program costs, including sampling and analytical techniques	http://immunizationeconomics.org/recent-activity/2019howtocost
Global Health Cost Consortium (GHCC)	GHCC Reference Case	2017	Health interventions in general	Improve quality of cost estimates	https://ghcosting.org/pages/standards/reference_case

DRAFT

Developer	Guidelines	Publication years	Target Interventions	Purposes	Link
WHO	Comprehensive Multi-Year Planning (cMYP): A Tool and User Guide for cMYP Costing and Financing	2014	Immunization Programs	To facilitate costing and finance estimation of a cMYP	https://www.who.int/immunization/programmes_systems/financing/tools/cmyp_costing_tool_manual.pdf
ICAN	Methodology note for systematic review, cost catalogue, and analytics<br><br>How to conduct the cost of a campaign: methodological guidance" to be completed in 2020*	2019	Immunization delivery costs	Designed for users of data, including national and sub-national planners and policymakers, researchers, and international partners supporting country immunization and health system policy, planning, and financing	http://immunizationeconomics.org/ican-idcc-methodology
IVI/WHO	CHOLTOOL User Guide	2015	Cholera-specific vaccination programs, including campaigns	Instructions for users of costing tools	https://www.tandfonline.com/doi/full/10.1080/21645515.2020.1747930
WHO	C4P tool: HPV Vaccination Modula User Guide	2012-2019	HPV vaccination programs	Instructions for users of costing tool	TBD
WHO	Flutool plus (SIICT): introduction planning and costing	2017	Seasonal influenza vaccination, including campaigns	Instructions for users of costing tool	https://www.who.int/immunization/research/development/Influenza_economics/en/

*This guide was published after the finalization of this consensus statement and can be found on the website (https://static1.squarespace.com/static/556deb8ee4b08a534b8360e7/t/61571c3d5bb7bb6ad93e720f/1633098822790/How+to+cost+an+immunization+campaign_29Sept.pdf).

Table A2b shows the characteristics of costing tools [6] that have been developed for costing vaccine delivery or immunization programs that were identified by the advisory group. It includes five tools for costing the introduction of single antigens, three to estimate immunization program costs, one for estimating the cost-effectiveness of introducing a new vaccine or vaccine technology, one for estimating vaccine technology costs

[6] Costing tools perform analysis and some have accompanying data forms such as the IVI CHOLTOOL

and health impact, and one for estimating costs of vaccination in the second year of life. Characteristics were self-reported by the tool developers on the advisory group.

Table A2b. List of costing tools for vaccine delivery or immunization program

	Delivery Modality	Antigens included	Retrospective vs. Cost projection data collection	Retrospective vs. projection analysis	Full or incremental costs	Economic vs. financial (or undepreciated financial)	Intended Perspective	Intended Data Sources	Sampling	Intended Use of Results
WHO C4P	Health facility; School; Multiple	HPV	Retrospective	Retrospective; Projection	Incremental	Economic; Financial; Initial Investment	Government; Provider; or Payer	Interviews; Financial records; Expert opinion	No guidance	Planning; RM; CEA
IVI CHOLTOOL	SIA/ campaign	Oral Cholera Vaccine	Retrospective	Retrospective; Projection	Incremental	Economic; Financial	Government; or Payer	Interviews; Financial records; Expert opinion	No guidance	Planning; RM; CEA
WHO SIICT	Health facility; SIA/ Campaign; Outreach; Multiple	Influenza	Retrospective	Retrospective Projection	Incremental	Economic; Financial	Government; Provider; or Payer	Interviews; Financial records; Expert opinion	No guidance	Planning; RM; CEA
WHO/IVI TCVCT	Health facility; SIA/ Campaign; Outreach; Multiple	Typhoid Conjugate	Retrospective	Retrospective; Projection	Incremental	Economic; Financial	Government; Provider; or Payer	Interviews; Financial records; Expert opinion	No guidance	Planning; RM; CEA
PATH MVICT	Health Facility; Outreach	RTS,S	Retrospective	Retrospective Projection	Incremental	Economic; Financial; Initial Investment	Government; or Provider	Interviews; Financial records; Expert opinion	No guidance	Planning; RM; CEA
WHO cMYP	Health facility; SIA/ Campaign; Outreach;	All	Retrospective	Retrospective Projection	Full	Undepreciated financial	Government	Interviews; Financial records; Expert opinion	No guidance	Planning; RM

DRAFT

	Delivery Modality	Antigens included	Retrospective vs. Cost projection data collection	Retrospective vs. projection analysis	Full or incremental costs	Economic vs. financial (or undepreciated financial)	Intended Perspective	Intended Data Sources	Sampling	Intended Use of Results
	Multiple									
UN OneHealth Tool	Health Facility; Outreach; Multiple	All	Retrospective Prospective	Projection	Incremental or Full	Financial	Government	Interviews; Financial records; Expert opinion	No guidance	Planning; Budgeting; RM; CEA
PAHO ProVac/ Costvac	Health Facility; Outreach	All	Retrospective	Retrospective	Full	TBD	Government Provider Payer	Interviews; Financial records; Expert opinion	Random selection; Convenience	Budgeting; Efficiency
PAHO ProVac/ UNIVAC	Health Facility/ Outreach	All	Retrospective	Retrospective	Full	TBD	Government Provider Payer	Interviews; Financial records; Expert opinion	Random selection; Convenience	Budgeting; CEA
PATH VTIA	Health Facility; Outreach	All	Retrospective	Projection	Incremental	Economic	N/A	Interviews; Financial records; Expert opinion	Convenience	Planning; Decision-making
UNICEF second year of life (2YL)	Health Facility	All	Retrospective	Projection	Incremental	Economic; Financial	Health sector Government	Interviews; Financial records; Expert opinion	No guidance	Planning

Abbreviations: C4P = Cervical Cancer Prevention and Control Costing (https://www.who.int/immunization/diseases/hpv/ cervical_cancer_costing_tool/en/); CHOLTool = Oral Cholera Vaccine Costing Tool; SIICT = Seasonal Influenza Immunization Costing Tool; TCVCT = Typhoid Conjugate Vaccine Costing Tool; MVICT = Malaria Vaccine Immunization Costing Tool; cMYP = Comprehensive Multi-Year Plan; 2YL = Second Year of Life; VTIA = Vaccine Technology Impact Assessment; RM = Resource Mobilization; CEA = Cost-Effectiveness Analysis

DRAFT

Annex 3. Definition of costing terminology

Table A3 shows the definition of costing terms presented in the various guidance documents. Most of the guidance documents have similar definitions of financial and economic costs, capital costs, and incremental costs but differ in the level of detail in their explanations. Fewer documents (less than three) have definitions of cost projections, prospective and retrospective costing, perspective, and bottom-up and top-down costing. The GHCC guidance document has the most definitions while other guidance documents focused on methods.

Other differences among the guidance documents are variations in definitions of vaccine delivery cost and prospective costing. The EPIC and ICAN definition of vaccine delivery are that it includes costs of delivering vaccines, exclusive of vaccines. The costing tools, however, use the term service delivery for operational costs of delivering vaccines, exclusive of vaccines, while 'vaccine delivery cost' includes all the value of all resources involved in the immunizations. Prospective costing is defined as 'direct observation' in EPIC and as projection of costs in the costing tools.

Table A3. Definitions of costing terms in guidance documents

	**WHO 1994**	**WHO 2002**	**ICAN & EPIC (including 'How to Cost Immunization Programs' and The Common Approach)**	**GHCC**	**Costing Tools' User Manuals**	**cMYP Guideline**	**Recommend ation**
**Vaccine delivery cost**	NA	NA	Costs associated with delivering immunizations to target populations, exclusive of vaccine costs (ICAN Methodology Note, pg.11)<br><br>All resources used, whether immunization-specific, or 'shared, and whether consumed at immunization delivery 'sites' or above the level of service delivery, with and	NA	Vaccine delivery includes startup costs, service delivery (personnel time, supplies and transport/allowance), vaccine procurement, monitoring and supervision, and other costs (C4P guide, pg. 262)	NA	Use ICAN/EPIC definition, specify whether is inclusive or exclusive of vaccines and that includes recurrent and capital costs.

	WHO 1994	WHO 2002	ICAN & EPIC (including 'How to Cost Immunization Programs' and The Common Approach)	GHCC	Costing Tools' User Manuals	cMYP Guideline	Recommendation
			without the new vaccine (How to cost immunization programs, pg. 4)				
**Financial cost**	NA	Actual expenditure for resources used for goods or services purchased. Does not include cost of existing health personnel time or donated goods (pg. 2)	Financial outlays, usually with straight-line depreciation of capital items (ICAN Methodology Note, pg. 31)<br>A financial costing is concerned with accounting transactions (i.e., monetary outlays or expenditures) (How to cost immunization programs, pg. 7)	Capture the resources that are 'paid' for (pg. A-8)	Actual monetary flows of the buyer such as the Ministry of Health. Does not include the value of resources already paid for, such as personnel time (SIICT guide, pg. 21)	NA	Composite of three definitions, noting that perspective affects the specification of the ingredients.
**Economic cost**	Value of resources used to produce something, including a specific health service or a set of services (pg. 13)	Resources that have been foregone for alternative uses, or opportunity costs (pg. 2)	A valuation of all inputs needed for the routine immunization program including valuation of time, supplies, equipment, and annualization of costs that adjusts for a discount rate (Common Approach, p. 6)<br><br>Financial outlays plus opportunity costs such as health worker time and any donated items such as vaccines (ICAN Methodology Note, pg. 56)	The value of the highest alternative health intervention opportunity forgone; captures the full value forgone of all resources used (pg. A-8)	Estimates all costs of an intervention, regardless of the source of funding, so that the opportunity cost of all resources is accounted for in the analysis, includes in-kind and donor contributions (SIICT guide, pg. 21)	NA	ICAN/EPIC definition, with clarification that includes resources from all payers/ resource providers.

	WHO 1994	WHO 2002	ICAN & EPIC (including 'How to Cost Immunization Programs' and The Common Approach)	GHCC	Costing Tools' User Manuals	cMYP Guideline	Recommendation
			An economic costing values resources based on their opportunity cost, regardless of whether a financial transaction occurred (How to cost immunization programs, pg. 7)				
**Undepreciated financial cost** (called initial investment in costing tool guides and referred to as fiscal costs in previous analyses)	NA	NA	Financial outlays, usually without depreciation of capital items (ICAN Methodology Note, pg. 31)	NA	Initial upfront resource requirements (C4P guide, pg. 268)	NA	ICAN/EPIC definition
**Start-up or introduction costs**	NA	NA	Costs that are incremental to the routine immunization system and specifically incurred as a result of introduction of the new vaccine (Common Approach, pg.17) All resources used for one-time activities (e.g., social mobilization, cold chain capacity mobilization expansion) in a defined time period around the	NA	Initial one-time programmatic activities and include micro-planning, initial training activities, and initial sensitization/ social mobilization/ IEC (SIICT guide, pg.21)	NA	Costing tool User Manual definition, with clarification on difference between initial and continuing activities

DRAFT

	WHO 1994	WHO 2002	ICAN & EPIC (including 'How to Cost Immunization Programs' and The Common Approach)	GHCC	Costing Tools' User Manuals	cMYP Guideline	Recommend ation
			introduction (How to Cost Immunization, pg. 4)				
**Recurrent cost**	NA	Items that are used up during a year (pg. 3)	Recurrent items include labor and consumable items such as vaccines doses, supplies and travel costs (How to cost immunization, pg. 11)	Value of resources/inputs with useful lives of less than one year (pg. 61)	Goods or items used in the delivery of a service or intervention that last less than a year, e.g., personnel salaries. (SIICT guide, pg. 21)	Costs of resources consumed within one year (CMYP guide, pg. 19)	Composite definition
**Capital cost (sometimes called investment cost)**	Inputs that last for more than one year (pg. 6)	Items that last longer than one year and are therefore incurred only every few years rather than annually (pg. 3)	Capital items are durable items such as building, equipment, and vehicles (How to cost immunization, pg. 11)	One-time costs for items that have a useful life of over one year (pg. B-23)	Goods that last for longer than one year, such as equipment (SIICT guide, pg. 21)	An input that has a useful life of more than one year (cMYP guide, pg. 19)	Composite definition
**Incremental cost**	NA	Only looks at the cost of an addition, e.g., a new vaccine, to existing services (pg. 2)	Additional costs associated with introducing new vaccines or making changes in delivery (ICAN Methodology Note, pg. 32)<br>Make assumptions about what particular resources were affected by the intervention, and only measure those resources (How to cost immunization, pg. 8)	Cost of adding a new or a batch of services or intervention over and above an existing program (pg. 59)	Additional resources required to add an intervention to an existing immunization program (CHOLTOOL guide, pg. 6)	NA	Composite of definitions, with clarification that if resources are not slack, then have to account of opportunity cost
**Full cost**	NA	NA	Full costs include baseline cost as well as the additional cost of the new intervention	NA	NA	NA	ICAN/EPIC definition, with clarification that includes vaccines and

	WHO 1994	WHO 2002	ICAN & EPIC (including 'How to Cost Immunization Programs' and The Common Approach)	GHCC	Costing Tools' User Manuals	cMYP Guideline	Recommend ation
			(How to cost immunization, pg. 8) The sum of all costs associated with vaccination delivery (ICAN Methodology Note, pg. 31)				basic infrastructure
**Prospective**	NA	NA	Direct observation (How to Cost Immunization Programs, pg. 21)	Direct observation of resource use (pg. B-18)		NA	Composite of definitions, with clarification that costs are collected concurrently with interventions implementation
**Retrospective**	NA	NA		Data collection takes place after resource use (pg. B-18)		NA	GHCC definition
**Cost projection**		NA	NA	NA	NA	Total future costs of both recurrent and capital inputs to the NIP (cMYP guide, pg. 108)	cMYP definition with clarification that is for intervention or program
**Micro-costing/Ingredi ents**	NA	NA	Approach in which prices and quantities of resources are measured (How to Cost Immunization Programs, pg. 4)	Focuses on granular accounting of inputs; Disaggregates costs of particular output into specific items consumed (pg. A-13)	NA	NA	GHCC definition
**Bottom-up Costing vs Top-down Costing**	NA	NA	NA	Bottom-up measures input quantities at the client or activity level; Top-down divides overall program cost	NA	NA	GHCC definition

DRAFT

	WHO 1994	WHO 2002	ICAN & EPIC (including 'How to Cost Immunization Programs' and The Common Approach)	GHCC	Costing Tools' User Manuals	cMYP Guideline	Recommendation
				or expenditures, often including those above service level, by number of outputs to calculate unit cost (pg. A-13)			
**Perspective**	NA	NA	The point of view considered for costs (and benefits, if included), in a costing study; to whom the costs were incurred. Common perspectives include provider, government, healthcare, insurer and societal. (ICAN Methodology Note, pg. 32) Perspective has to do with which costs we care about. A study from the "societal" perspective should include all costs, no matter who in society pays them. The more commonly used "health sector" perspective is narrower. (How to Cost Immunization Programs, pg. 7)	Describes which payers' costs are included in the estimate. For example, a provider perspective may include costs incurred by health service providers, non-health service providers, and be limited to specific payers. (pg. B-2)	NA	NA	Composite definition

Sources: WHO 1994 Training Manual (https://apps.who.int/iris/handle/10665/400300); WHO Cervical Cancer screening and treatment module user manual (https://www.who.int/immunization/diseases/hpv/cervical_cancer_costing_tool/en/); ICAN (http://immunizationeconomics.org/ican-idcc-methodology); GHCC (https://ghcosting.org/pages/standards/reference_case); WHO 2013 cMYP

DRAFT

Guidelines (https://www.who.int/immunization/programmes_systems/financing/tools/cmyp/en/#:~:text=cMYP%20Guidelines&text=Better%20alignment%20of%20immunization%20and,than%20by%20disease%20or%20initiative)

In addition, most guides define incremental costing but not full costing. Only one of the documents (EPIC) had nuanced discussions of how perspective affects financial costs, incremental costing affects economic costs, and how the purpose of the analysis affects what cost ingredients should be included. Specifically, the perspective of the costing affects the designation of which inputs/resources are donated – e.g. vaccines in GAVI-eligible countries are donated if the perspective is the government and therefore would appear only as an economic cost, whereas if the study were conducted from a health sector perspective these might be included as financial costs. It is critical to clarify if the perspective is defined in terms of the payer (i.e., the organization outlaying the funds directly to the provider of goods or services) or the funding source; for example, when donor funds are channeled to the government and the government conducts the monetary outlay, this would be considered both an economic cost if the government perspective is used but a financial and economic cost if defined in terms of the payer (donor). Thus, the perspective will affect which resources are included in financial costs. For incremental costs, the guides define these as additional costs incurred with the introduction of a new vaccine or other technology but don't indicate what inputs/resources should be included in economic costs – i.e., which recurrent and existing capital costs should be included.

DRAFT

Annex 4. Costing principles

Table A3 compares the costing principles in the guidance documents with the GHCC Principles and Methods Reporting Checklist. The guidance documents focus on five of the principles: 1) defining the purpose of the study (GHCC principle 1); 2) classifying the costs as recurrent/capital and financial/economic (GHCC principle 3) ; 3) specifying the time horizon of data collection (GHCC principle 5) ; 4) presenting costing methods (GHCC principle 7); and 5) depreciating the capital costs (GHCC principle 12).

Other GHCC principles were only discussed in one or two of the other guidance documents: 1) importance of stating the perspective (GHCC principle 2); 2) scope of costing (GHCC principles 5 and 6); 3) sampling strategy (GHCC principle 8); 4) timing of data collection (GHCC principle 10); 5) sources for price data (GHCC principle 11); 6) selection of discount rate (GHCC principle 13); 7) use of shadow prices (GHCC principle 14); and 8) characterization of uncertainty (GHCC principle 16). The recommended costing principles are also found in Section 5 of the Consensus Statement.

Table A4. Comparison of costing principles among guidance

GHCC Principle #	WHO 1994	EPIC/Common Approach/Reference Guide	Costing Tools	cMYP	Recommendation in Consensus Statement (CS) (Section 5)
**1** The purpose, the population, and the intervention and/or service/output of the cost estimation should be clearly defined.	NA	At the earliest stage of planning a costing exercise, one should consider objectives and rationale.	User should assess whether financial or economic costs are most appropriate based on the objective (C4P, SIICT, CHOLTOOL, SIICT, TCV, MVICT)	The objectives are to analyze program costs, financing and financing gaps and these should be linked to the program objectives.	Combined GHCC principles 1 and 5 (CS Principle # 2): The study scope in terms of its purpose, audience, target population, time horizon, and service/output should be clearly stated. It should also state whether data collection will be prospective or retrospective, and whether the analysis will be retrospective or a cost projection.
**2** The perspective of the cost estimation should be stated and justified.	NA	Perspective is an important concept that is somewhat unique to economic studies, as compared to other types of health service research.	NA	NA	Applied GHCC principle (CS Principle # 3): The perspective of the cost estimation should be stated and justified.

GHCC Principle #	WHO 1994	EPIC/Common Approach/Reference Guide	Costing Tools	cMYP	Recommendation in Consensus Statement (CS) (Section 5)
**3** The type of cost should be clearly defined, in terms of economic vs. financial, incremental vs full cost, and whether the cost is 'net of future cost.'	Costs should be classified by inputs: recurrent and capital; Can also be classified by function/activity, level, source, and type of currency; Economic costing should be used for cost-effectiveness analyses.	It is important to make the distinction between financial and economic costs.	Costs are classified as financial and economic as well as recurrent and capital in the costing tools. (C4P, SIICT, CHOLTOOL, TCV, MVICT)	Costs are defined as recurrent and capital.	Composite of definitions(CS Principle # 4): Types of costs to be generated should be clearly defined in terms of startup/ introduction or non-startup/introduction, recurrent and capital, undepreciated financial, financial or economic, and incremental or full. Capital costs should be appropriately annualized and depreciated for financial and economic costs and the discount rate justified.
**4** The 'units' in the unit costs for strategies, services and interventions should be defined.	Explains general nature of unit costs and gives examples of unit costs.	All resources used in an intervention divided by number vaccination	Unit costs are measured as cost per dose administered, child or girl fully vaccinated	NA	Composite of definitions (CS Principle # 6): The 'units' in the unit costs for strategies, services and interventions should be defined – e.g., cost per dose administered or cost per FIC.
**5** The time horizon of data collection should be explicit and of sufficient length to capture costs relevant to the purpose, and consideration should be given to disaggregating costs into separate time periods where they vary over time.	Should choose the most recent year for which cost data are available for one full year.	When collecting primary data retrospectively, one must set boundaries of the time horizon in which resource use occurred.	The user should specify whether the estimates are cost projection or retrospective analyses. (C4P, CHOLTOOL, MVICT)	Planning horizon is five years or less.	Combined GHCC principles 1 and 5 (CS Principle # 2)
**6** The scope of the inputs to include in the cost estimation should be defined and justified relevant to purpose.	Need to be clear about scope of the costing.	The decisions about scope should be made when planning the exercise, before data is collected.	NA	NA	Composite of definitions (CS Principle # 5): The scope of the inputs to be estimated should be defined, justified and if needed referenced. For example, do the costs include

DRAFT

GHCC Principle #	WHO 1994	EPIC/Common Approach/Reference Guide	Costing Tools	cMYP	Recommendation in Consensus Statement (CS) (Section 5)
					national and sub-national costs or only facility service delivery costs? Are non-immunization costs included?
**7** The methods for estimating the quantity of inputs should be described, including methods, data sources and criteria for allocating resources.	NA	Presents methods for recurrent and capital costs.	Presents methods of calculation and suggests data sources. (C4P, SIICT, CHOLTOOL, TCV, MVICT)	Ingredients approach is used to estimate costs – quantities x price x % used in immunization.	Composite of definitions (CS Principle # 9): The methods for estimating the quantity of inputs should be described – whether top-down or bottom-up, methods of allocation, use of shadow prices and opportunity cost of time, and, methods for excluding research and evaluation costs.
**8** The sampling strategy used should be specified and designed to minimize bias.	It is necessary to choose a sample and use one of four types: either random, cluster, systematic, or stratified.	Published guidance for sampling health facilities that was developed for health facility data collection alongside DHS household surveys can be applied to immunization costing studies.	NA	NA	Combined definitions and edits by advisory group (CS Principle # 12): The sampling strategy employed should aim for internal and external validity of the data. Sampling strategy should be stated, described, and justified, depending on the workstream and costing objectives. Sampling of different service delivery units is desirable as it provides a more representative picture of costs and highlights cost variation and cost drivers for a strategy or vaccine.
**9** The selection of the data source(s) and methods for estimating service use should be described, and potential	Methods are described.	Recommend being aware of the quality of available data sources and reporting systems and comparing data sources.	Data sources and methods for estimating service use are described. (C4P, SIICT,	NA	Composite of definitions (CS Principle # 8): The selection of the data sources, including any adjustments to price data (e.g., inflation or currency

DRAFT

GHCC Principle #	WHO 1994	EPIC/Common Approach/Reference Guide	Costing Tools	cMYP	Recommendation in Consensus Statement (CS) (Section 5)
biases reported in the study limitations.			CHOLTOOL, TCV, MVICT)		conversion) should be described and referenced.
**10** Consideration should be given to the timing of data collection to minimize recall bias and, where relevant, the impact of seasonality and other differences.	NA	Notes that the major advantage of direct observation methods is lack of recall bias.	NA	NA	Not included
**11** The sources for price data should be listed by input, and clear delineation should be made between local and international price data sources, and tradeable, non-tradeable goods.	NA	The Common Aoproach lists the sources of information for unit vaccine prices. The HOW TO COST… document lists sources of data for prices.	Sources for price data should be noted in the designated worksheets**.** (C4P, SIICT, CHOLTOOL, TCV, MVICT)	NA	Included in CS Principles # 8 and 9
**12** Capital costs should be appropriately annuitized or depreciated to reflect the expected life of capital inputs	Recommends straight line depreciation.	For economic cost evaluation, all capital costs need to be annualized based on a discount rate and estimates of useful life.	Straight line depreciation is calculated for financial costs, and annualization and discounting for economic costs. (C4P, SIICT, TCV, MVICT)	NA	Included in CS Principle # 4
**13** Where relevant, an appropriate discount rate, inflation and exchange rates should be used, and clearly stated.	NA	Recommends using a 3% discount rate unless there is another justification.	NA	NA	Include in CS Principle # 4
**14** The use and source of shadow prices for goods and the opportunity cost of time should be reported.	NA	NA	NA	NA	Include in CS Principle # 9
**15** Variation in the cost of the intervention by site/organization, sub-populations, or by other drivers of heterogeneity	NA	One of the main questions of the Common Approach is to assess the factors that drive the variation between facility total and unit costs. EPIC has	NA	NA	Included in CS Principle # 13

DRAFT

GHCC Principle #	WHO 1994	EPIC/Common Approach/Reference Guide	Costing Tools	cMYP	Recommendation in Consensus Statement (CS) (Section 5)
should be explored and reported.		an analytical tool and database to facilitate comparisons across facilities.			
**16** The uncertainty associated with cost estimates should be appropriately characterized.	NA	Standard statistical approaches can be used to calculate an unbiased measure of mean, and the uncertainty in this mean estimated.	NA	Recommends scenario-building to take in account uncertainty; also risk assessment.	Combined two principles in CS Principle 14: The uncertainty around the cost estimates should be appropriately characterized,(e.g., sensitivity analyses; ranges of results for different input parameter scenarios for cost projections; mean and standard deviation for non-representative samples with multiple units; and confidence intervals or credible intervals for retrospective analyses.
**17** Cost estimates should be communicated clearly and transparently to enable decision-maker(s) to interpret and use the results.	NA	Section in the Common Approach focuses on writing up results	NA	It is essential to communicate the results clearly.	Combined two principles in CS Principle # 16: Cost estimates should be communicated clearly and transparently to enable decision-makers to interpret and use the results.

DRAFT

Annex 5. Characteristics of costing workstreams

Table A5 shows characteristics of the four costing workstreams identified based on recent work known to the advisory group. It shows that the activities/cost categories used in costing are largely similar within the guidance documents for immunization costing. However, in a few cases, the terminology differs – e.g. vaccines/injection supplies for program costing, vaccine procurement for cost projections and retrospective campaign costing, and vaccine, collection, distribution and storage for retrospective routine immunization costing. In addition, some workstreams use the term service delivery to encompass health personnel time, supplies, and transport while other workstreams separate these into individual components. Also, two of the workstreams, program costing and retrospective routine costing, explicitly mention surveillance as an activity while the other workstreams include surveillance under the monitoring activity/cost category. Similarly, two of the workstreams include micro-planning, cost projections and retrospective campaign costing, while this activity is not included in the other workstreams.

Table A5. Characteristics of costing workstreams

	**Level of Data Collection**	**Activities/Cost categories**	**Perspective**	**Incremental or full**	**Similarities and Differences in workstream guidance in definitions of terms and perspective**
Retrospective routine immunization cross-sectional costs	Facility with some data collection at higher levels	Vaccine (procurement), collection, distribution, storage<br>Facility-based service delivery (personnel. time and resources)<br>Monitoring and evaluation<br>Supervision<br>Training<br>Social mobilization<br>Surveillance<br>Program management<br>Cold chain maintenance<br>Other capital	Provider, Payer, or Societal	Full or Incremental	- Similar definitions of financial and economic costs and recurrent and capital costs<br>- Uses health sector perspective
Retrospective single-vaccine costs	Program and facility with sampling or interviews with program managers	Vaccine procurement<br>Service Delivery (personnel and transport)<br>Distribution<br>Supervision<br>Micro-planning<br>Training<br>Other Recurrent<br>Cold Chain	Provider, Payer, or Societal	Incremental	- Similar definitions of financial and economic costs and recurrent and capital costs<br>- Uses government and payer perspectives<br>- Costing tools assume incremental economic costs do not include existing equipment

	Level of Data Collection	Activities/Cost categories	Perspective	Incremental or full	Similarities and Differences in workstream guidance in definitions of terms and perspective
		AEFI Surveillance<br>Other capital			since these have available capacity (excess capacity)
Projection of new vaccine introduction costs	Program and facility	Vaccine procurement<br>Service Delivery (personnel and transport)<br>Distribution<br>Supervision<br>Micro-planning<br>Training<br>Other Recurrent<br>Cold Chain<br>AEFI Surveillance<br>Other capital	Provider, Payer, or Societal	Incremental	- Similar definitions of financial and economic costs and recurrent and capital costs<br>- Uses government and payer perspectives<br>- Assumes incremental economic costs do not include existing equipment since these have available capacity (excess capacity)
Projection of immunization program costs	Program	Vaccines/injection supplies<br>Personnel<br>Transport<br>Social Mobilization/IEC<br>Training<br>Supervision<br>Monitoring (includes surveillance<br>Cold chain equipment<br>Other capital	Provider (could include external funding)	Full or Incremental	- Similar definitions of recurrent and capital costs except for US$100 requirement for capital costs per item; uses straight line depreciation<br>- Cost projections also similar to other definitions<br>- Perspective is government but includes value of donated goods and personnel time

Variation among Workstreams

The workstreams shows the different approaches on data sources, sampling, and characterization of uncertainty, as shown in Table A6. This makes sense given the different recommended uses of the different workstreams. For example, cost projections of new vaccine introduction or an five-year immunization program are by definition an exercise in assumptions about an unknown future program with hypothetical information on costs and quantities; therefore, larger or more representative sampling of sites may not reduce uncertainty about this future program, whereas exploration of a range of scenario input parameters can help identify influential programmatic and cost elements and the range of possible cost results.

DRAFT

Table A6. Data sources, sampling and characterization of uncertainty, and terminology by workstreams

	Recommended Use	Perspective	Data Sources	Sampling	Characterizing Uncertainty
Retrospective routine immunization cross-sectional costs	Compare costs of vaccine delivery for benchmarking and to explain variation in facility costs and unit costs and evaluate efficiency and equity	Provider, Payer, or Societal	Health facility records; interviews with national and sub-national program managers	Representative sampling of health facilities (stratified, random)	Characterized based on number of sites in sample, stratification of units, and basis of probability of selection; one-way sensitivity testing or scenario analysis
Retrospective single-vaccine costs	Estimate costs of campaigns or routine health facility delivery for benchmarking and to explain variations by strategy and venue	Provider payer, or Societal	Interviews with national and sub-national program managers	Representative sampling of health facilities or campaign sites; Convenience samples	Characterized based on number of sites in sample, stratification of units, and basis of probability of selection
Projection of new vaccine introduction costs	Estimate costs to assist program managers in planning and decision-making on vaccine introduction	Provider, payer, or Societal	No guidance provided; Practice is to use expert opinion; conduct visits to selected health facilities; and hold workshops with stakeholders	No guidance provided	Costing tools are not specific but suggest use of scenarios
Projection of Immunization program costs	Estimate costs to assist in budgeting, planning and resource mobilization over a five-year period	Provider	Interviews with national and sub-national program managers; visits to selected health facilities sometimes	Can collect data at the sub-national as well as national levels	Conduct scenario analysis to have a range of estimates

DRAFT

Annex 6. Areas for clarification and harmonization

Areas for clarification and harmonization are defined as problem areas or areas without a consensus. The following are the areas that have been identified from the review of guides and costing tool manuals.

1. Definitions on terminology among and within workstreams differ and need to be harmonized, where appropriate, acknowledging the different workstream purposes. See Annex 6 for recommended terms
2. The options for study perspective should be agreed upon by advisory group, including use of perspective in financial vs. economic costing.
3. Inconsistent labeling of program activities vs. resource inputs as cost categories, inconsistent nesting of resource inputs inside program activities and vice versa without regard for the perspective of the analysis.
4. Definition of incremental and full costing is not consistent.
5. Sampling and uncertainty: What are the appropriate sampling approaches (random, purposive) for different costing objectives (assuming time and money are not the limitations)? What level of uncertainty is appropriate?
6. Gaps in practical guidance on aggregating costs across levels of the health system and clarity on level of activity vs. level of payer.

DRAFT
